# “Visiting scientist effect”? Exploring the impact of time‐lags in the digitization of 2D landmark data

**DOI:** 10.1002/ar.25649

**Published:** 2025-03-31

**Authors:** Andrea Cardini

**Affiliations:** ^1^ Dipartimento di Scienze Chimiche e Geologiche Università di Modena e Reggio Emilia Modena Italy; ^2^ School of Anatomy, Physiology and Human Biology The University of Western Australia Crawley Western Australia Australia

**Keywords:** data collection, geometric morphometrics, measurement error, precision, Procrustes

## Abstract

Measurement error (ME) in geometric morphometrics has been the subject of countless articles, but none specific to the effect of time lags on landmark digitization error. Yet, especially for visiting scientists working on museum collections, it is not uncommon to collect data in multiple rounds, with interruptions of weeks or years. To explore the impact of time lags on Procrustes shape analysis, I repeatedly digitized the same landmarks, on photographs of crania of adult yellow‐bellied marmots, at progressively longer time intervals, ranging from a few hours to days, weeks and, in one case, many years. Using a battery of methods, I found that there is indeed a time‐related systematic ME suggesting the possibility of a “visiting scientist effect” biasing shape patterns. However, the relationship between time lags and the magnitude of the bias is not simple and linear, but complex. Interestingly, the impact of the bias on the results of tests of sexual dimorphism and allometry is modest, and mostly negligible, unless the design of the data collection is highly unbalanced. When this happens, as in a simulated case where females are digitized first and males only later (or vice versa), the effect of the bias on tests of biological variation becomes important and can even lead to opposite conclusions on group differences. I will discuss when systematic ME in landmark data is more problematic and how to try to mitigate the impact of a potential “visiting scientist effect” on shape analyses.

## 1 INTRODUCTION

Geometric morphometrics (GMM) is a family of methods for the quantitative analysis of biological forms (Adams et al., 2004; O'Higgins, 1997). Among them, those using the Cartesian coordinates of anatomical landmarks and a Procrustes superimposition to separate size and shape are the most widely applied (Adams et al., 2004), including in integrative taxonomy (Cardini, 2024a, 2024b). Morphometric analyses often deal with subtle differences that require accurate measurements. For instance, they can be used to compare cryptic species, which may be supported by molecular evidence without any obvious morphological divergence (Dujardin et al., 2010; Karanovic et al., 2016; Mahmoudi et al., 2017). Furthermore, they can help to measure clinal variation in closely related populations (Cardini et al., 2007; Frost et al., 2003; Monteiro et al., 2003), and quantify fluctuating asymmetry, the often minute random differences between mirror sides of symmetric organisms (Klingenberg et al., 2002). Especially when the variation being measured is small, carefully assessing measurement error (ME) is crucial. Indeed, what the sources of ME are in GMM and the best approaches for its assessment have been the subject of a large number of methodological papers and reviews (Arnqvist & Martensson, 1998; Bakkes, 2017; Cardini, 2014, 2024a; Cardini et al., 2022; Collyer & Adams, 2024a; Engelkes et al., 2019; Evin et al., 2020; Fox et al., 2020; Fruciano, 2016; Fruciano et al., 2017; Galimberti et al., 2019; Klingenberg et al., 2002; Messer et al., 2021; Olsen et al., 2023; Shearer et al., 2017; von Cramon‐Taubadel et al., 2007; Vrdoljak et al., 2020; Wasiljew et al., 2020). Yet, it seems that, despite the attention given to ME in the literature, even recent GMM studies seldom assess ME (Cardini, 2024a). Besides, even if a variety of aspects of ME in GMM has been considered, nobody seems to have yet explored the impact of time lags in data collection on ME. In fact, Evin et al. (2020) mentioned that this might be an important issue, but it was not part of their analysis or any other I could find in the English GMM literature.

It is not uncommon for morphometricians to collect data over a relatively long time period. This is especially frequent when large data collections are done by visiting several museums. To provide a few examples, I will be borrowing from my own experience of data collection on mammals. My largest morphometric data collection happened in 2004. We needed a large sample for a series of studies on craniofacial variation in cercopithecine monkeys (Cardini et al., 2007; Cardini & Elton, 2008). Thus, that year, in approximately 7 months, I measured with a Microscribe 3D digitizer (https://gomeasure3d.com/microscribe/) about 100 anatomical landmarks directly on the skulls of almost 4000 monkeys. In that period, I visited, sometimes one after the other and sometimes with a few weeks of interruption, some of the largest natural history museums in Europe and the United States. A few years later, as we expanded the study to cranial variation in African colobines (Cardini & Elton, 2009; Nowak et al., 2008), I measured several hundreds more specimens, which mostly originated from the collection of the Royal Museum for Central Africa. Thus, the total dataset contained measurements taken in various rounds of landmark digitization, separated by time lags that ranged from a few days to weeks or even, for the very last set of measurements on colobines, to years. Back then, although with rather crude methods, we explored the magnitude of ME in both cercopithecines (Cardini & Elton, 2008) and colobines (Nowak et al., 2008), and found it to be negligible. However, we never investigated the potential effect of longer time lags on ME. Luckily, cranial differences between the two subfamilies are so large that, even when analyzed together, it is unlikely that a time‐related bias (i.e., a directional or systematic ME^1^) may impact results. However, if analyses were conducted within genera or species, where variation is small, one should not rule out a potential effect of time lags during data collection. To exemplify this type of effect, I will be using another example from my own work. In this second case, unfortunately, the consequence of a long separation between two main periods of data collection was clear and impactful.

**FIGURE 1:**
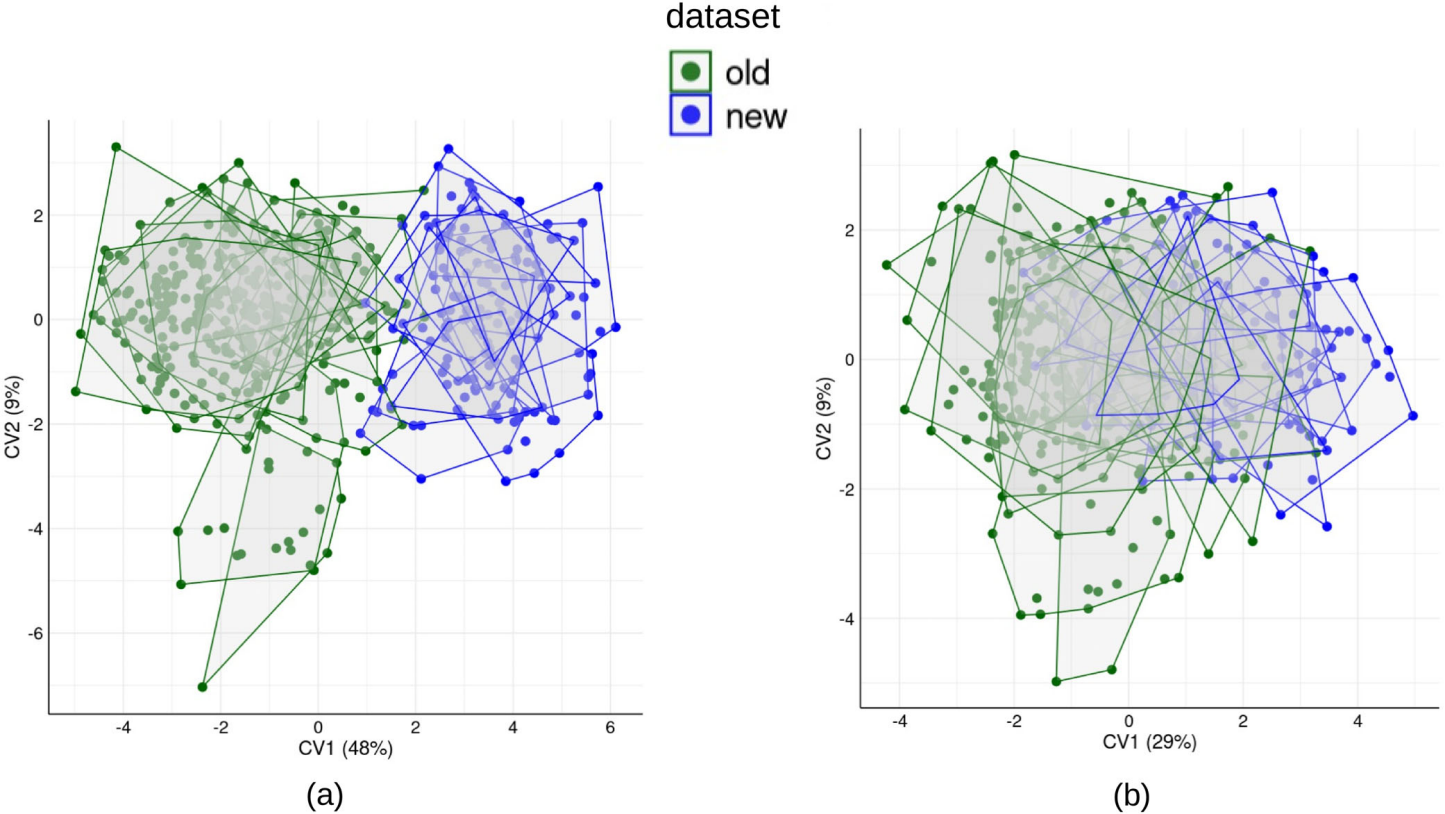
Examples of bias due to data collection time lag: scatterplot of CV1‐2 (between group variance in parentheses) from a CVA of 3D cranial shapes in a large sample of African adult men: (a) using the first 46 PCs (80% of total variance) of the Procrustes shape coordinates of all 92 cranial landmarks, (b) using the first 46 PCs (99% of total shape variance) of a subset of 33 “more precise” landmarks. Convex hulls show the 32 geographic populations whose differences are being maximized in the CVAs; green and blue symbols, respectively, show the data collected first (old dataset) and years later (new dataset).

In the same years of the data collection on Old World monkeys, I was also cooperating with anthropologists interested in the morphological variation of humans across sub‐Saharan Africa (Franklin et al., 2010). A large cranial dataset of adult men had gaps in its geographic coverage, so a second round of digitizing was done to include specimens that could fill these gaps. When the shape data from the two rounds of digitizing were merged, it became immediately obvious that the separation between groups, as evident from a Canonical Variates Analysis (CVA) of the geographic populations (Figure 1a), did not correspond to the distinction between geographic populations but rather to the year of data collection. This is surprising because neither the operator nor measurement instrument (a 3D Microscribe) had changed. Even when we selected a subset of landmarks easier to be precisely located, such as, for instance, some of the intersections of well visible sutures, a degree of separation between older and newer data remained (Figure 1b). Most likely, it was the time‐lag of a few years, separating the two periods of data collection, that had introduced a directional error large enough to bias the pattern of shape variation. After a long time since the previous round of digitization, even a well trained and experienced operator might tend to see the position of the landmarks slightly differently, with some landmarks more strongly affected than others. This explanation seems more parsimonious than alternatives, as the digitizer was not damaged during the transportation and its accuracy, in fractions of a millimeter, should not impact measurements on objects as large as an adult human cranium. In the end, because we had no chances to obtain a second digitization of at least a subsample of the specimens measured in the first, older, round of data collection, and, thus, use it to explore and maybe correct the bias, we had no other option but to discard the data and abandon the study. The unfortunate incident, however, left me wondering whether and how often this type of systematic error, that I nickname the “visiting scientist effect,” may happen. The occurrence of time lags, when samples are measured, is hardly ever mentioned in GMM articles, but it is probably not rare that morphometric data are collected at different times by one or several operators. In the second case, a possible “visiting scientist effect” will be almost certainly aggravated by systematic differences between operators (Engelkes et al., 2019; Fox et al., 2020; Fruciano, 2016; Fruciano et al., 2017; Messer et al., 2021; Shearer et al., 2017; Vrdoljak et al., 2020).

**FIGURE 2:**
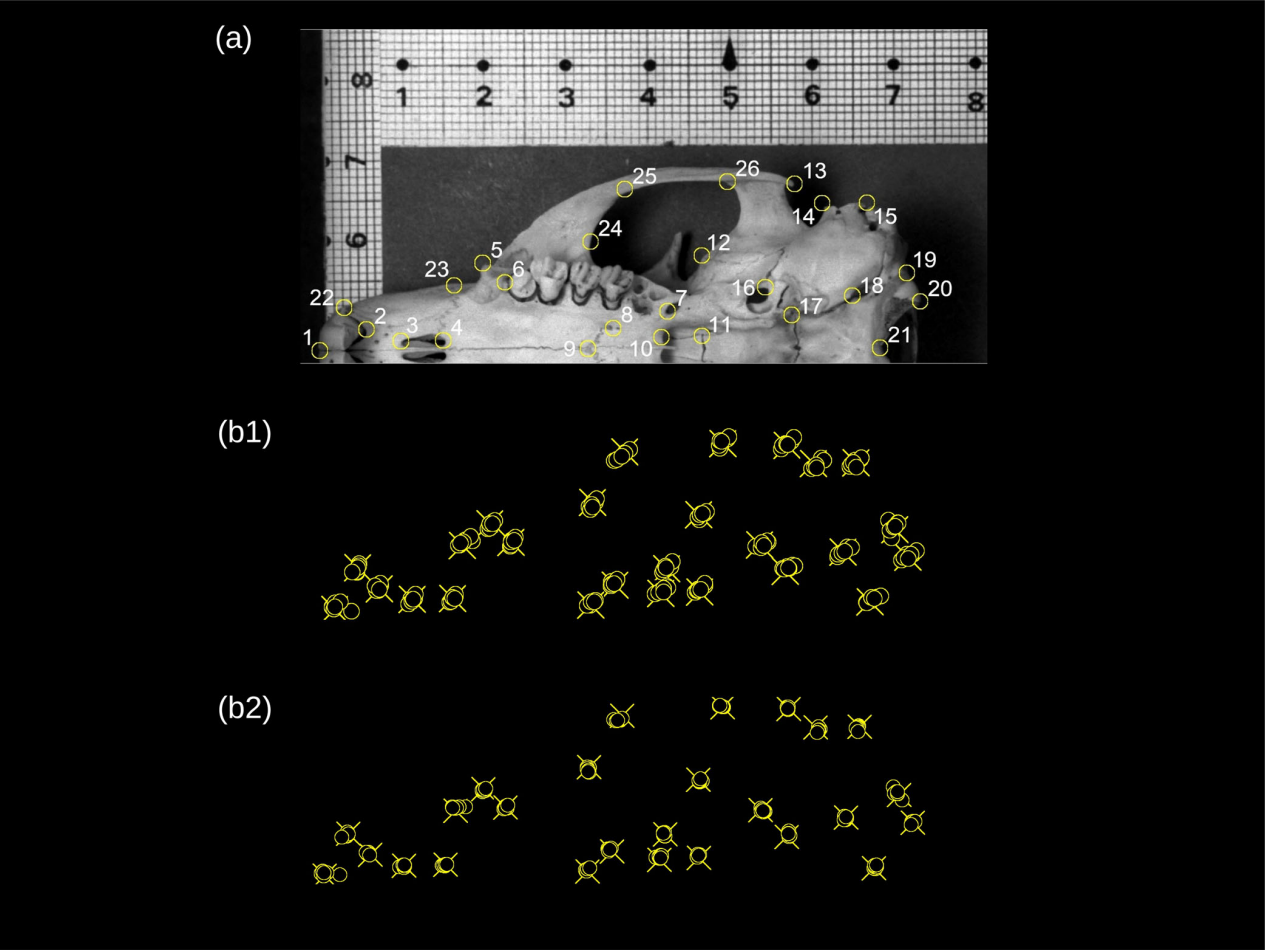
(a) Landmark configuration (modified from Cardini & O'Higgins, 2004). Landmark definition (modified from the same reference): L1 Anterior (midsagittal) tip of the premaxilla. L2 Posterior extremity of the incisor alveolus. L3–4 Extremities of incisive foramen. L5 Tip of the masseteric tubercle. L6 Anterior extremity of the toothrow. L7 Posterior maxillary foramen. L8 Posterior palatine foramen. L9 Suture between maxilla and palatine along the midsagittal plane. L10 Point of maximum curvature on the posterior edge of the palatine. L11 Meeting point between basisphenoid and presphenoid where the anterior foramen lacerum typically opens. L12 Anterior extremity of the suture between the alisphenoid and the zygomatic process of the squamosal. L13 Posterior tip of the zygomatic arch. L14–15 Anterior and posterior tip of the external auditory meatus. L16 Posterior extremity of the foramen ovale. L17 Meeting point between the basisphenoid, basioccipital, and tympanic bulla. L18 Anterior extremity of the jugular foramen. L19–20 Lateral tips of the occipital condyle. L21 Most posterior point on the ventral region of the occipital foramen. L22 Upper extremity of the incisor alveolus. L23 Most lateral point of the rostrum along the suture between the premaxilla and the maxilla 24 Most anterior point of the orbit (in the ventral view). L25 Marked change in curvature along the anterior region of the upper internal side of the zygomatic arch. L26 Anterior region of the squamosal zygomatic process where it joins the zygomatic arch. (b) An example of repeated digitization (REPs) of the same individual before (b1) and after (b2) superimposition, highlighting the first recent REP (d0001a; Table 1) by marking its landmarks with crosses, in contrast to the circles used for all the other REPs. For this individual, the centroid size (CS) ranged from 116 to 119 mm, with a median of 118 mm.

In this study, I want to explore whether time lags in the digitization of landmark data increase ME and potentially introduce a systematic error that could alter the results of GMM analyses. To this aim, I am using 2D data (i.e., photographs) of the ventral side of adult yellow‐bellied marmots (*Marmota flaviventris* (Audubon & Bachman, 1841)). On these photographs, which I had landmarked 20 years ago and used in previous research (Cardini & O'Higgins, 2004), I have re‐digitized the same 26 landmarks (Figure 2) repeatedly, with digitizations separated by progressively longer time lags. However, because landmarks were re‐digitized on the same photographs, instead of repeating all the steps of the data collection (repositioning the crania, taking at least two photographs, etc.), as desirable for a really accurate assessment of total ME (Arnqvist & Martensson, 1998), only digitizing error is investigated. Yet, for brevity, I will refer to digitization error in this study simply as ME. This partial assessment, that excludes all other sources of error (position, camera, 2D approximation of a 3D structure, etc.), is a limitation. The true total ME is underestimated. However, there are some advantages. The interpretation of results is easier by focusing on a single source of error. Also, as the items being measured are in exactly the same position in all digitizations of each individual, it is possible to measure the absolute per‐landmark precision of the raw data (i.e., the Cartesian coordinates of the landmarks as they are measured on the photographs, before being Procrustes superimposed—see Methods—to obtain shape variables).

**FIGURE 3:**
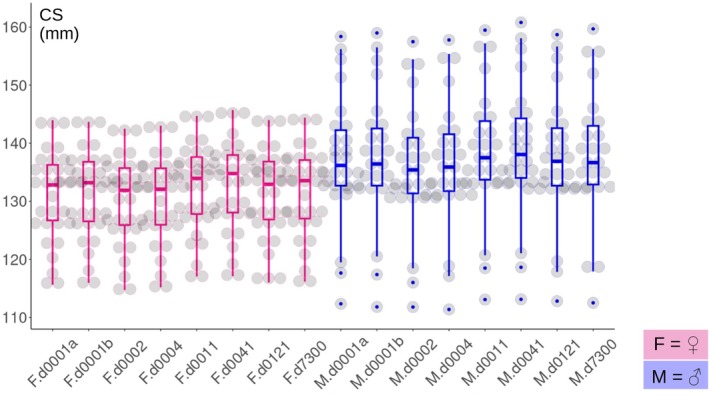
Box and jitter‐plot of cranial centroid size (CS) variation within each REP split by sex (females = pink; males = blue).

In the analysis, I employed multiple approaches. The main method was the recently developed ME ANOVA (Collyer & Adams, 2024a), which is specifically tailored to detect biases. The study only considered shape. This is not because shape is more interesting than size. They are both important (Cardini, 2020). However, it has been shown that size (specifically centroid size in Procrustean GMM) is typically much less strongly affected by imprecision in landmark digitization (Cardini, 2024a, and references therein). This conclusion was backed by preliminary analyses (not shown) in the current dataset. These showed that individual variation in cranial size of yellow‐bellied marmots, based on the 26 landmarks, accounted for more than 100 times more variance than that explained by differences among repeated digitizations (i.e., ME). Accordingly, a box and jitter‐plot of cranial size (Figure 3) suggested very similar patterns of variation across all digitization repetitions (REPs), with no clear trend of increasing differences over time lags. Thus, because size is not analyzed, and all analyses are done exclusively on shape, I might often report or discuss analyses without specifying every time that they only concern shape. In brief, using Procrustes shape variables (Adams et al., 2004), the main study aims were:

1. Testing whether there was a systematic ME (or bias) in REPs of the same landmarks and exploring whether imprecision increased in relation to the time lag between digitizations.
2. Assessing the robustness of the inferences and patterns in aspects of biological shape variation, such as sexual dimorphism and allometry, across REPs, despite landmark imprecision.
3. Finding the most imprecise landmarks in absolute terms using the raw data.
4. Assessing whether the exclusion of highly imprecise landmarks appreciably reduced the impact of ME on shape analysis, including the potential effect of data collection time lags.

## 2 MATERIALS AND METHODS

### 2.1 Data

tThe sample consisted of 58 crania (32 females and 26 males) of adult yellow‐bellied marmots (*Marmota flaviventris* (Audubon & Bachman, 1841)) from the collection of the US Natural History Museum in Washington DC. Photographs of ventral crania were taken in standardized settings, as detailed in previous studies (Cardini & O'Higgins, 2004; Cardini & Tongiorgi, 2003).

The original, first digitization of 2004 (~20 years; hence, ~7300 days ago) was used in this analysis as the oldest REP. Since the time lag of this digitization was so old, I plotted it as the last in the time series in the results and graphics, even though it was the first one conducted. The advantage of this expedient is that the data were presented in relation to increasingly longer time lags. All other REPs were more recent and were done between the end of 2023 and the beginning of 2024, within approximately 4 months since the first digitization, using progressively longer time lags. All digitizations were done in TPSDig (Rohlf, 2015). As an acronym for discriminating the eight digitizations (in total), I used d (day) followed by the number of the day on which the landmarks were digitized. Table 1 summarizes all the acronyms used in this paper.

**TABLE 1:** Main abbreviations used in the study, including the other tables.

Abbreviation	Definition
ANOVA	Analysis of variance, which for shape is multivariate
CS	Centroid size
bgPCA	Between group principal component analysis
d0001a,b; d0002; …; d0121; d7300	Day 1 first (a) or second (b) repeat; repeat in day 2; then, always counting from the first digitization, repeats in day 4, 11 (i.e., 1 week after the previous one), 41 (1 month after the one in day 11), 121 (i.e., 3 months after day 41). d7300, however, is the original digitization of approximately 20 years ago (365 by 20 = 7300 days ago), which is the one with the longest time gap and, thus, is placed as if it was the last in the series.
df	Degrees of freedom
F, M	Respectively, females and males
GMM	Geometric morphometrics (in this study, narrowly used to refer to landmark‐based Procrustes methods)
HR	Leave‐one out cross‐validated classification accuracy of females and males using shape in a bgPCA
L	Landmark: e.g., L1, L2, L3, etc.
ME	Measurement error, which in this paper is landmark digitization error and only concerns imprecision
SS and MS	Respectively, sum of squares and mean sum of squares
systME	Systematic ME or bias (both terms are used interchangeably in the article)
randME	Random ME
totME	Total ME (the sum of systematic and random ME)
PCA	Principal component analysis
REP	Repetition of digitization
Rsq	Multivariate variance accounted for by a factor
SNR	Signal to noise ME ratio (e.g., variation accounted for by systematic ME divided by variation accounted for by random ME)
UPGMA/NN	Respectively, unweighted pair group method with arithmetic mean and nearest neighbor (single linkage) methods for building shape phenograms

Before each digitization, the order of the specimens was randomized in TPSUtil (Rohlf, 2015). Digitizations were done twice on day 1 (d0001a, in the morning, and d0001b, in the afternoon), then on day 2 (d0002), day 4 (d0004), day 11 (d0011), day 41 (d0041) and day 121 (d0121), with the last three REPs being respectively 1 week, 1 month, and 3 months after the previous one. The last REP in the series was, in fact, as anticipated, the oldest, with an approximate time lag of 7300 days (d7300). Thus, overall, there were eight REPs for a total of 464 digitizations on the same 58 photographs (i.e., 12,064 digitized landmarks).

### 2.2 Geometric morphometrics

For the analyses of shape, centroid size (CS) was calculated as the square root of the sum of squared distances between the landmarks and their centroid (i.e., the mean point in the X Y raw data space) and configurations were scaled to unit size by dividing the raw coordinates of each digitization by the corresponding CS. Positional differences were minimized using a Procrustes superimposition (Gower, 1975; Rohlf & Slice, 1990; Sneath, 1967) to compute the Procrustes shape coordinates.

Not to distract readers later, I anticipate here a result concerning a secondary issue in the context of the analysis of Procrustes shape coordinates. An Euclidean tangent space approximation of the curved Procrustes shape space is necessary for most statistical analyses, which require the data space to be Euclidean. As typical of most GMM studies (Marcus et al., 2000), the approximation in this dataset was excellent, with pairwise Procrustes shape distances (the metric measuring shape differences) in the curved shape space virtually identical to the corresponding Euclidean distances in the flat tangent plane (r = 1, slope = 1 in an origin‐centered regression, of the two types of distances, done in TPSSmall; Rohlf, 2015).

### 2.3 Preliminary considerations on statistical analyses: Organization and terminology, significance threshold and R square, summaries of multivariate shape

I subdivided the section on statistical methods into four subsections (M1, M2, M3, M4). Likewise, results will be presented subdivided into four parts (R1, R2, etc.), which correspond to the four sets of statistical shape analyses.

This study mostly concerns precision or repeatability. Repeatability refers to the ability to repeat the results of a study using the same measurements on the same subjects by the same protocol; precision refers to how close the measurements are to each other and accuracy how close they are to the true value. For shape analysis in Procrustean GMM, there is a caveat to bear in mind. Because, with Procrustes superimposition methods (Gower, 1975; Rohlf & Slice, 1990; Sneath, 1967), shape variables are obtained using a biologically arbitrary but statistically convenient coordinate system (Adams et al., 2004, and references therein), the Procrustes shape coordinates must always be analyzed all together with multivariate techniques (Rohlf, 1998). This also implies that any analysis, interpretation, or visualization of shape variation one landmark at a time is meaningless (Cardini & Verderame, 2022). However, because in my study REPs were done on the same photographs and only used to assess digitization error, there were no positional differences between REPs of the same individuals. Therefore, beside assessing ME in shape using multivariate statistics, I could also explore per‐landmark differences in raw coordinates among REPs, as explained below (M3). As a convention in this article, I will refer to this type of variation in the raw coordinates due to digitization error as landmark “absolute precision” (or, for its opposite, as “absolute imprecision”).

This study makes extensive use of tests of null hypotheses. However, not to rely only on P values (the probability of the data to be compatible with the null hypothesis—Greenland, 2019; Greenland et al., 2016), I report also the magnitude of the effect (i.e., the factor) being tested. As an estimate of effect size, I used multivariate R square (Rsq), which is the proportion of multivariate variance in shape accounted for by the factor being tested. Rsq is a biased estimator, which tends to be inflated in small samples (Cramer, 1987), but it is intuitive and easy to interpret (Cardini, 2024a, 2024b). In this study, when a test was repeated (e.g., ANOVAs using full or reduced landmark configurations or selected pairwise comparisons—see 2.4), the sample size (N) remained the same. Besides its simplicity, Rsq has other advantages in the context of this study, whose main aim is to explore a possible time lag‐related trend in ME and its practical impact on biological hypotheses:

1. Rsq is the only effect size statistic available in all the programs used in this study (see 2.8). This can be seen as a limitation of some of these programs, but the use of multiple programs has the advantage of running tests in independently developed software and can be used to double‐check the results.
2. Rsq can be reported for all variance components, including random ME.
3. Rsq is consistent with the test statistics used to detect systematic ME (see 2.4), which is the ratio between variance accounted for by systematic ME and variance accounted for by random ME. Thus, the Rsq of the different ME components has a simple direct relationship to the test statistics being used.
4. For the same reasons, Rsq is consistent with the eigenvector plots (EV—see 2.4) used by Collyer and Adams (2024a) to visualize biases using the EV of the matrix product of the systematic ME test statistics.
5. Rsq is used in all the sciences and tens of thousands of published studies, using univariate or multivariate statistical methods (for a crude idea, a Google Scholar search in October 2024 for “coefficient of determination,” which is the common name for Rsq, produced 5,760,000 entries).

As a threshold for statistical significance, I consistently employed 0.005. This is lower than the conventional 0.05, but it has been suggested as appropriate whenever reducing the risk of type I errors (rejecting the null hypothesis when true) is more important than statistical power (Benjamin et al., 2017). Both thresholds are, nonetheless, arbitrary and cannot be used with a simplistic binary approach, as *P*‐values represent a continuum that must be carefully interpreted (Greenland et al., 2016). In this study, although several comparisons were planned a priori (e.g., the first REP vs. each of the other REPs), the same hypothesis was often tested multiple times (e.g., using different landmark configurations or repeating the test of sex or allometric variation). Thus, there might be inflations of type I errors, which were partially reduced by adopting a lower significance threshold. Moreover, morphometric data on mammal crania, which typically belong to specimens collected in an opportunistic way, are likely to be autocorrelated and plagued by biased sampling in museum collections (Cardini, 2020, 2024a), which suggests additional caution before confidently rejecting a null hypothesis.

Procrustes shape data tend to be highly dimensional. In my dataset, with 26 2D landmarks, there were 52 Procrustes shape coordinates, although they have a degree of redundancy and the real number of informative dimensions is 48 (52 minus four degrees of freedom lost in the superimposition—Rohlf & Slice, 1990). To summarize shape variation, I used two main methods. One is a principal component analysis (PCA) using the variance–covariance matrix of the Procrustes shape coordinates. The other is a cluster analysis on the matrix of the sample pairwise Procrustes shape distances. A PCA is an ordination method and, thus, produces summary scatterplots, which allow maximizing variance in fewer, statistically orthogonal axes (the principal components or PCs). A cluster analysis produces summaries in the form of trees (phenograms), where branch lengths are proportional to differences. As algorithms for the cluster analysis, I used either a UPGMA (unweighted pair group method with arithmetic mean) or an NN (nearest neighbor or single linkage) method.

### 2.4 M1: ME tests and time‐related bias assessment

I employed a parametric Procrustes ANOVA (Klingenberg et al., 2002; Viscosi & Cardini, 2011) with sex as the main factor and individual as the random factor to test whether mean sex differences are significantly larger than individual variation and whether the latter is significantly larger than differences between REPs of each individual (i.e., the estimate of total ME). Because the analysis is hierarchical, the order of the factors matters. Having sex first, as a main factor, implies that mean sex differences are removed before individual variation is compared to total ME. If the grouping variable of interest is sex, this may seem inappropriate, because individual variation is reduced by statistically controlling for sexual dimorphism. Indeed, this step is optional and represents a conservative approach to the assessment of differences between “true biological” variation in sample and ME. However, the approach that includes main group factors before assessing individual differences in relation to ME has advantages. Mainly, it offers some protection against the risk that any within‐group test one might need to perform (e.g., separate search for outliers within females or males, tests of differences between sexes in allometric trajectories, etc.) could be impacted by ME becoming relatively larger (Cardini, 2024a).

The Procrustes ANOVA is not specific to ME, with one of its main applications being in the context of the analysis of symmetry and asymmetry (Klingenberg et al., 2002, and references therein). This statistical technique, although frequently employed to explore ME (Fruciano, 2016; Viscosi & Cardini, 2011), does not allow detecting biases due to a consistent pattern of imprecision in landmark digitizations. By bias in the digitization of a landmark, I mean that, for instance, I might have unintentionally but systematically placed, in most images of a given REP, L16 slightly to the left of the posterior extremity of the foramen ovale, where it should be. Thus, in this REP, L16 will be on average closer to the center of the foramen, in a position that is biased compared to other REPs. A random digitization error, in contrast, means that I randomly misplaced a landmark (slightly to the left or right, above or below, etc.) with no consistent direction in the imprecise position where it is digitized.

A new ANOVA design, called ME ANOVA (Collyer & Adams, 2024a), has been recently and specifically developed to separate the two types of ME, systematic and random. I provide here a simplified explanation of the approach, without formulas, and refer readers to the original paper for an extensive and rigorous mathematical treatment of the subject. With this method, individual means are subtracted from each individual's own REP values, and systematic ME is estimated, with some similarities to a repeated measures ANOVA (Fruciano, 2016, and references therein), by the average difference between REPs. For instance, if I had 10 individuals and two REPs, first I would compute the deviations of each individual from the mean of its two REPs to control for individual variation; later, using these individual “mean‐centered” data, I would compute the mean of the 10 individuals in the first REP and in the second REP, and finally statistically compare these two averages. This way, the bias is tested in relation to random ME, which is the individual deviations from the mean of the REP of each individual. The idea is that, with the effect of individual variation controlled for, the bias in the data (systematic ME) should not be appreciably larger than noise (random, non‐directional, ME). In this context, Collyer and Adams refer to the ratio between the variances of these two components of ME as a signal to noise ratio (SNR), which they use as a test statistic. I stress that the use of “signal” in this context deviates from the common use in reference to the specific biological question one is interested in (e.g., sex or species differences, allometry, etc., as, for instance, in Cardini, 2024a). In the ME ANOVA, the main signal the researcher wants to detect is the bias in the REPs. Individual variation is also tested, but as with systematic ME, individual differences (the signal in this specific test) are compared to random ME (the noise), rather than being compared to total ME, as in Procrustes ANOVA. Additionally, the ME ANOVA uses type II sum of squares (SS), which differs from the Procrustes ANOVA in MorphoJ, where type I SS is used. As a result, in Procrustes ANOVA, the sum of the variances explained by all factors always totals 100%, while with ME ANOVA, the total Rsq values may not exactly sum to 100%.

When there are groups, the ME ANOVA can also assess the interaction between systematic ME and groups. If the interaction is significant, it means that the bias varies among groups. Thus, I applied the ME ANOVA including sex as a factor. Besides testing the different components of ME, the ME ANOVA, as it is implemented in RRPP (Collyer & Adams, 2024b), produces EV plots, where mean‐centered individual data of all REPs in the analysis are projected onto the eigenvectors, which maximize systematic ME variance relative to random ME variance. EV plots, therefore, are visual summaries of variation in the subspace of ME. This means that the proportion of variance accounted for by each EV only refers to systematic ME variance. This is analogous to between‐group PCA (bgPCA) scatterplots (Cardini et al., 2019, and references therein), which focus only on the fraction of total multivariate variance that distinguishes groups, a fraction that can vary in size depending on the case. Also, like in a bgPCA, the number of bgPCs equals the number of groups minus one; in a REP EV plot, the number of EV axes is the number of REPs minus one. With two REPs, there will be just one EV axis accounting for 100% of systematic ME variance. With eight REPs, as in the main ME ANOVAs of my dataset, there will be seven EV axes, with EV1 accounting for most of the systematic ME variance, EV2 for the second largest systematic ME variance, and so forth. Altogether, the seven EV axes account for 100% of ME variance, but I stress it again, this is only the fraction of total shape variance due to systematic ME. Thus, with, for instance, two REPs, EV1 accounts for 100% of systematic ME variance, but that might be, say, just 1% of total shape variance.

I did the ME ANOVAs not only using all REPs together, but also with seven planned pairwise comparisons of selected REPs to assess ME in relation to increasingly long time lags between digitizations. Thus, the first of the most recent REPs (d0001a) was tested against the second (d0001b), third (d0002), and so forth. REP, with the last comparison being the one of d0001a with the oldest REP (d7300), that corresponds to the longest time lag (many years compared to hours, days, weeks or months in the recent REPs). The expectation was that, if ME increases with time between landmark digitizations (the “visiting scientist effect”), it should be tiny in REPs done in the same day (d0001a and d0001b) and, then, become progressively larger (Rsq) and more significant, as time increases. If the operator tends to see most landmarks in a slightly but systematically different position, as time goes by, another prediction is that systematic ME should show a stronger “visiting scientist effect” compared to random ME, which might remain relatively constant.

### 2.5 M2: Robustness of the findings across REPs

ME can impact results of analyses so that, if the same analysis is repeated in different REPs, results may be different. This may (as in the made‐up example in the next sentence) or may not happen. For instance, if there were two REPs and the second one had a much larger random ME compared to the first one, when sex differences are tested, results may differ between REPs. This is because within group variance is inflated in the second REP, so that sexual dimorphism could be masked and, thus, even if significant in the first REP, it could be non‐significant in the second one. A simulated, and rather extreme, but didactically useful example of this type of random ME masking group differences can be found in Figure 5c of Cardini (2024a). Systematic ME, in contrast, should not alter results if it is similar across groups in all REPs. Thus, for instance, the test of sex differences in shape should produce a similar output in the first, second, third, and so forth. REP both in terms of Rsq and P value. However, going on with the simple example of just two REPs, if the bias is stronger in males, and only in males, of REP1, but similar in both sexes of REP2, the estimate of sexual dimorphism could be inflated (or, less likely, deflated, depending on the direction of the bias) in REP1 compared to REP2.

In order to explore the impact of imprecision in landmark digitization on results of shape analyses, I used mean sex differences and static allometry (with pooled sexes) as examples of biologically interesting variation that a morphometrician may want to study. Static allometry refers to the covariation of shape with size within a structure in a specific age class of a species (Klingenberg, 2016); in my dataset, this applies to adult yellow‐bellied marmots. Allometry is interesting in itself (Klingenberg, 1998, 2016), but also because, sometimes, controlling for allometry, when groups (sex, taxon, etc.) are compared, might provide further insight into the nature of their differences (Cardini, 2024b). Thus, I repeated tests of shape sexual dimorphism and static allometry in each REP. I did both tests using a multivariate regression of shape (all shape coordinates) onto a dummy variable for sex (coding females as zero and male as one) or, for static allometry, onto CS. The statistical significance of the effect being tested was assessed using 999 permutations of the data in the full multivariate shape space. This test can be performed in MorphJ or vegan (*adonis2* function), as well as in other R packages. The implementation is slightly different, as MorphoJ uses Rsq (the variance “explained” by sex or CS) as a test statistic and *adonis2* employs an F test based on sequential SS from permutations of the raw data, but results in the two programs are generally equivalent. For sex differences, I also computed the leave‐out cross‐validated average classification accuracy (average hit rate—HR) of the a priori groups (i.e., the 32 females and 26 males) using all shape variables in a bgPCA. This method is equivalent to a discriminant analysis (DA) or (when used as an ordination method) canonical variate analysis (CVA), as it uses multivariate distances in the data space to classify individuals in relation to the closest group average. However, unlike a DA/CVA, a bgPCA is done without transforming the predictors (i.e., in my case, the Procrustes shape coordinates) and, therefore, employs Procrustes shape distances directly measured in the full Procrustes shape space (Rohlf, 2021, and references therein). If sex differences in ventral cranial shape are large in yellow‐bellied marmots, not only should sex be significant (with a large R‐squared value), but the average classification accuracy (HR) should also be high and closer to 100% rather than the 50:50 expectation for two groups of approximately similar sample sizes.

I also explored the congruence of patterns and similarity relationships in shape data across REPs. For patterns, I used PC1 within each REP as a proxy for the main variation trend in the shape data and pairwise computed the absolute Pearson correlation (r) of PC1 scores between any two REPs. The absolute correlation, which captures the strength of the association between the two variables, was employed because the sign of a PC in a PCA is arbitrary. For the overall congruence of shape data, I pairwise computed the matrix correlations between individual Procrustes shape distances calculated within each REP. As with r for PC1, the matrix correlation measures the proportionality of individual differences between the two datasets. For both types of correlations, I summarized the pairwise comparisons using their median and 10th and 90th percentiles.

### 2.6 M3: Raw coordinates per‐landmark variance (absolute imprecision) and detection of imprecise landmarks

To assess absolute per‐landmark imprecision in the raw coordinates, I computed the variance among the eight REPs of the X coordinates of landmark one (L1) for the first individual in the sample, repeating the process for the Y coordinates. Finally, I summed them up and used the sum of the X and Y variances to estimate absolute imprecision for L1 in the first individual of the sample. Similarly, I computed the raw coordinates variance of L1 for individual two, three, and so forth. To summarize absolute L1 imprecision, I calculated the variance's sample median, 90th percentile, mean, and standard deviation (SD). I repeated the entire process for all the landmarks.

Having calculated the summary statistics for per‐landmark variances of each landmark, to graphically summarize results, I focused mainly on medians and 90th percentiles, as they should be less strongly affected by outliers. Therefore, I re‐ordered landmarks according to increasing medians of per‐landmark variances and used a profile plot of all four summary statistics to visualize the trend in absolute imprecision. As in Cardini (2024a), I also “standardized” medians and 90th percentiles of per‐landmark variances by the corresponding medians of these two summary statistics. The resulting standardized statistics are easier to interpret, as they re‐express results in terms of how many times a landmark is on average less precise compared to all others. For example, a standardized median of 0.5 indicates that the landmark is half as imprecise as the average of all 26 landmarks, while a standardized median of 1.5 means it is 50% more imprecise than the average. With 90th percentiles, the rationale is the same. To combine these two, generally highly congruent (see Results) standardized summary statistics, I simply summed them up to obtain a single value for each landmark. Then, in the plots of per‐landmark variances, I color‐coded the landmarks using a gradient from blue (highest precision) to red (highest imprecision), with the color tone proportional to the sum of standardized medians and 90th percentiles of per‐landmark variances.

As a visual aid to explore and interpret absolute imprecision in raw landmark coordinates, I also computed for each landmark and each individual the deviation of the raw coordinates from the mean of its eight REPs. These deviations quantify in each individual, and for each landmark, how a landmark was displaced in a digitization compared to the average of its REPs. Thus, when the deviations of all individuals are added to the raw coordinates of the corresponding landmarks of an individual, one can easily see the scatter in the landmark configuration, which is purely due to absolute imprecision^2^ (Cardini, 2024a). In this graph, which I called the “absolute per‐landmark imprecision” plot, the choice of the specific individual to add the deviations is arbitrary but irrelevant: using a different individual does not change the patterns of scatter of the points around the position they have in that or any other specimen.

### 2.7 M4: Assessment of the exclusion of imprecise landmarks on overall ME and bias

Using the results of M3, I set two arbitrary thresholds for high imprecision and used the landmarks below the thresholds to subset the total configuration and explore the impact on ME of removing the most imprecise landmarks. The first threshold defined the most imprecise landmarks as those above 1.5 on both axes in the scatterplot of standardized medians and 90th percentiles of per‐landmark raw coordinates variances (see M3, above). These are landmarks that are more than 50% less precise than average using both summary statistics. I called “precise configuration” the subset of landmarks resulting from the exclusion of the most imprecise ones. I also defined a second more stringent subset of landmarks, called the “most precise configuration,” by excluding all landmarks above one for both the standardized medians and 90th percentiles of per‐landmark variances (i.e., all those above average absolute imprecision in the total configuration). I stress that the thresholds were arbitrary and the analyses using more precise landmarks were largely exploratory. Also, “precise” and “most precise” are clearly relative concepts in relation to the specific data of the eight REPs. Even the most precise landmarks, if, for instance, the operator is inexperienced or very sloppy in the digitization, could have very large imprecision.

To assess whether using “precise” and “most precise” configurations impacted the ME estimates, the tests of mean sex differences and static allometry, and the congruence of shape data across REPs, I repeated on these two configurations all the analyses described in 2.4 (M1) and 2.5 (M2) on these two configurations. The impact on ME may seem obvious; however, it may not be the case. For instance, absolute imprecision in raw landmark coordinates may be unrelated to systematic ME in shape and only impact random ME or vice versa, or, albeit less likely, the impact could be negligible on both components of shape ME.

### 2.8 Software

I performed most analyses in R 4.4.1 (R Core Team, 2024), using a range of packages and custom scripts, with some of the code modified from the one written by ChatGPT (OpenAI, 2022), accessed in June–July 2024. The main software/packages in the study were:

1. MorphoJ 1.08.02 (Klingenberg, 2011), and the packages Morpho 2.12 (Schlager, 2017), geomorph 4.0.8 (Adams et al., 2024), and RRPP 2.0.3 (Collyer & Adams, 2024b) for the Procrustes superimposition and the main GMM analyses (see below). To avoid redundant references for these programs, I will use the software names without repeating the version and reference. Specifically, I conducted the parametric Procrustes ANOVA in MorphoJ and the permutational (999 permutations) ME ANOVA in RRPP.
2. ggplot2 (Wickham, 2016) was used for the box and jitter plot and some of the scatterplots.
3. ape 5.0 (Paradis & Schliep, 2019) and TreeView 1.6.6 (Page, 1996) were employed for tree editing and drawing.
4. Morpho *groupPCA* function was used for the cross‐validated classification of females and males, using a bg PCA.
5. The vegan (Oksanen et al., 2024) *adonis2* function was employed for testing mean shape sex differences and static allometry using permutations (999) of Euclidean distances.
6. Morpheus et al. (Slice, 1999) was used for shape diagrams using wireframes and deformation grids (Klingenberg, 2013).

Other R functions, such as those used to compute PCAs, phenograms, correlations, and so forth. belong to the base and stats packages of R (R Core Team, 2024).

## 3 RESULTS

A brief exploratory check for strong outliers in phenograms (UPGMA and NN using Procrustes shape distances) showed two evident outliers (both in REP d0041). Implementing the procedures outlined in Cardini (2024a), the “find outliers” option of MorphoJ confirmed the relatively large shape distances to the sample average of these two observations, likely due to the misplacement of L17 in one case and L25 in the other. However, since the misplacement was a consequence of a digitization error, and thus relevant to this assessment, these specimens were not excluded from the analysis.

### 3.1 R1: ME Tests and time‐related bias assessment, including all landmarks

In all ANOVAs, individual variation (Rsq >80%) was significantly larger than the total (Procrustes ANOVA) or random (ME ANOVA) ME for all landmarks and REPs (Table 2). In contrast, sexual dimorphism in the Procrustes ANOVA was small (Rsq ≈2% on average) and statistically negligible compared to the individual differences. However, ME ANOVAs demonstrated a bias in the REPs (Tables 2 and 3). The bias was small in terms of Rsq (2.2% using all REPs [Table 2]; ranging from 0.2% to 3% in pairwise tests between d0001a and each of the other REPs [Table 3]) and similar for both sexes (non‐significant interaction between sex and systematic ME; Rsq *r* ≤0.3%). Nonetheless, in the SNR tests, which relate systematic ME to random ME, the systematic bias was virtually always significant.

**TABLE 2:** Procrustes ANOVA (parametric) and ME ANOVA (999 permutations).

	Procrustes ANOVA									ME ANOVA						
Dataset	Effect	df	SS	MS	F	P(F)	Pillai trace	P(Pillai)	Rsq	Effect	df^a^	SS	MS	SNR	P(SNR)	Rsq
All landmarks	Sex	48	0.0065	0.000136	1.3	0.0766	0.9	0.1046	1.9%							
	Individual	2688	0.2795	0.000104	34.6	*<0.0001*	29.3	*<0.0001*	81.1%	Individual	57	0.2808	0.00493	5.64	*0.001*	81.4%
										systME	7	0.0077	0.00111	0.16	*0.001*	2.2%
										systME*sex	7	0.0011	0.00015	0.02	0.029	0.3%
										randME	392	0.0498	0.00013			14.4%
	totME	19,488	0.0586	0.000003					17.0%							
	Total		0.3447							Total	463	0.3450				
All landmarks without d7300	Sex	48	0.0057	0.000118	1.3	0.0955	0.9	0.0383	1.9%							
	Individual	2688	0.2476	0.000092	31.7	*<0.0001*	29.6	*<0.0001*	82.0%	Individual	57	0.2487	0.00436	5.72	*0.001*	82.3%
										systME	6	0.0042	0.00069	0.10	*0.001*	1.4%
										systME*sex	6	0.0010	0.00016	0.02	0.023	0.3%
										randME	336	0.0435	0.00013			14.4%
	totME	16,704	0.0486	0.000003					16.1%							
	Total		0.3018							Total	405	0.3021				
Precise landmarks	Sex	38	0.0082	0.000216	1.5	0.0207	0.8	0.1752	2.3%							
	Individual	2128	0.3008	0.000141	40.0	*<0.0001*	24.9	*<0.0001*	82.8%	Individual	57	0.3023	0.00530	6.31	*0.001*	83.1%
										systME	7	0.0057	0.00081	0.12	*0.001*	1.6%
										systME*sex	7	0.0009	0.00013	0.02	0.200	0.3%
										randME	392	0.0479	0.00012			13.2%
	totME	15,428	0.0545	0.000004					15.0%							
	Total		0.3635							Total	463	0.3638				
Most precise landmarks	Sex	22	0.0121	0.000549	1.8	0.0172	0.4	0.5287	2.5%							
	Individual	1232	0.3861	0.000313	34.2	*<0.0001*	14.8	*<0.0001*	80.5%	Individual	57	0.3886	0.00682	5.19	*0.001*	80.9%
										systME	7	0.0056	0.00080	0.07	*0.001*	1.2%
										systME*sex	7	0.0014	0.00020	0.02	0.346	0.3%
										randME	392	0.0748	0.00019			15.6%
	totME	8932	0.0817	0.000009					17.0%							
	Total		0.4799							Total	463	0.4805				

*Note*: Empty cells correspond to effects that are tested in one but not the other ANOVA (e.g., sex in the Procrustes ANOVA but not in the ME ANOVA), while effects present in both analyses are placed on the same row for comparison. In this and the following tables, abbreviations are as listed in Table 1; the most important results for the Discussion are highlighted with a light gray background, and significant tests (*p* < 0.005) are emphasized in underlined italics.

^a^ The apparent mismatch in degrees of freedom (df) between the two ANOVAs arises because the Procrustes ANOVA is parametric and must account for the 48 shape variables, following the loss of four df during the superimposition. In contrast, the df in the ME ANOVA are irrelevant for permutation tests, so they are presented as if the analysis were univariate. To compare the two, the df of the ME ANOVA must first be multiplied by 48. For example, with all landmarks, the df for total ME in Procrustes ANOVA is 19,488. In the ME ANOVA, the df for ME components (7 + 7 + 392 = 406) multiplied by 48 also equals 19,488, matching the Procrustes ANOVA.

**TABLE 3:** ME ANOVA pairwise between d0001a and every other REP: Summary results showing Rsq, SNR and P for the factors in the analysis.

			d0001a versus						
Dataset	Statistic	Factor	d0001b	d0002	d0004	d0011	d0041	d0121	d7300
All landmarks	Rsq	Individual	90.2%	89.2%	90.2%	89.0%	90.3%	89.5%	87.8%
		systME	0.2%	1.4%	0.7%	0.8%	0.9%	0.9%	3.0%
		systME*sex	0.2%	0.2%	0.3%	0.3%	0.2%	0.2%	0.2%
		randME	8.3%	8.1%	7.8%	9.0%	7.5%	8.2%	7.9%
	SNR	Individual	10.8	11.0	11.5	9.9	12.1	10.9	11.1
		systME	0.02	0.17	0.09	0.09	0.12	0.11	0.38
		systME*sex	0.03	0.03	0.04	0.03	0.02	0.03	0.02
	P(SNR)	Individual	*0.001*	*0.001*	*0.001*	*0.001*	*0.001*	*0.001*	*0.001*
		systME	0.165	*0.001*	*0.001*	*0.001*	*0.001*	*0.001*	*0.001*
		systME*sex	0.084	0.049	0.006	0.025	0.104	0.035	0.182
Precise landmarks	Rsq	Individual	91.3%	90.0%	91.1%	90.0%	91.3%	90.5%	90.2%
		systME	0.2%	0.8%	0.4%	0.6%	0.6%	0.7%	1.7%
		systME*sex	0.1%	0.1%	0.1%	0.2%	0.1%	0.1%	0.1%
		randME	7.2%	7.9%	7.1%	8.1%	6.7%	7.5%	6.7%
	SNR	Individual	12.6	11.4	12.8	11.1	13.6	12.1	13.4
		systME	0.03	0.10	0.05	0.07	0.09	0.09	0.25
		systME*sex	0.02	0.01	0.02	0.03	0.02	0.02	0.02
	P(SNR)	Individual	*0.001*	*0.001*	*0.001*	*0.001*	*0.001*	*0.001*	*0.001*
		systME	0.051	*0.001*	*0.001*	*0.001*	*0.001*	*0.001*	*0.001*
		systME*sex	0.237	0.697	0.274	0.009	0.534	0.651	0.542
Most precise landmarks	Rsq	Individual	89.4%	87.5%	89.0%	89.5%	89.7%	89.3%	89.8%
		systME	0.1%	1.1%	0.5%	0.6%	0.5%	0.5%	0.8%
		systME*sex	0.2%	0.2%	0.2%	0.3%	0.2%	0.1%	0.2%
		randME	9.0%	9.8%	9.0%	8.3%	8.2%	8.7%	7.8%
	SNR	Individual	10.0	8.9	9.9	10.8	10.9	10.3	11.5
		systME	0.02	0.12	0.05	0.07	0.06	0.06	0.10
		systME*sex	0.02	0.02	0.02	0.03	0.02	0.02	0.02
	P(SNR)	Individual	*0.001*	*0.001*	*0.001*	*0.001*	*0.001*	*0.001*	*0.001*
		systME	0.553	*0.001*	*0.002*	*0.001*	*0.001*	*0.001*	*0.001*
		systME*sex	0.484	0.505	0.267	0.015	0.314	0.535	0.302

*Note*: The light gray background is here used to emphasize results for the systematic ME.

**FIGURE 4:**
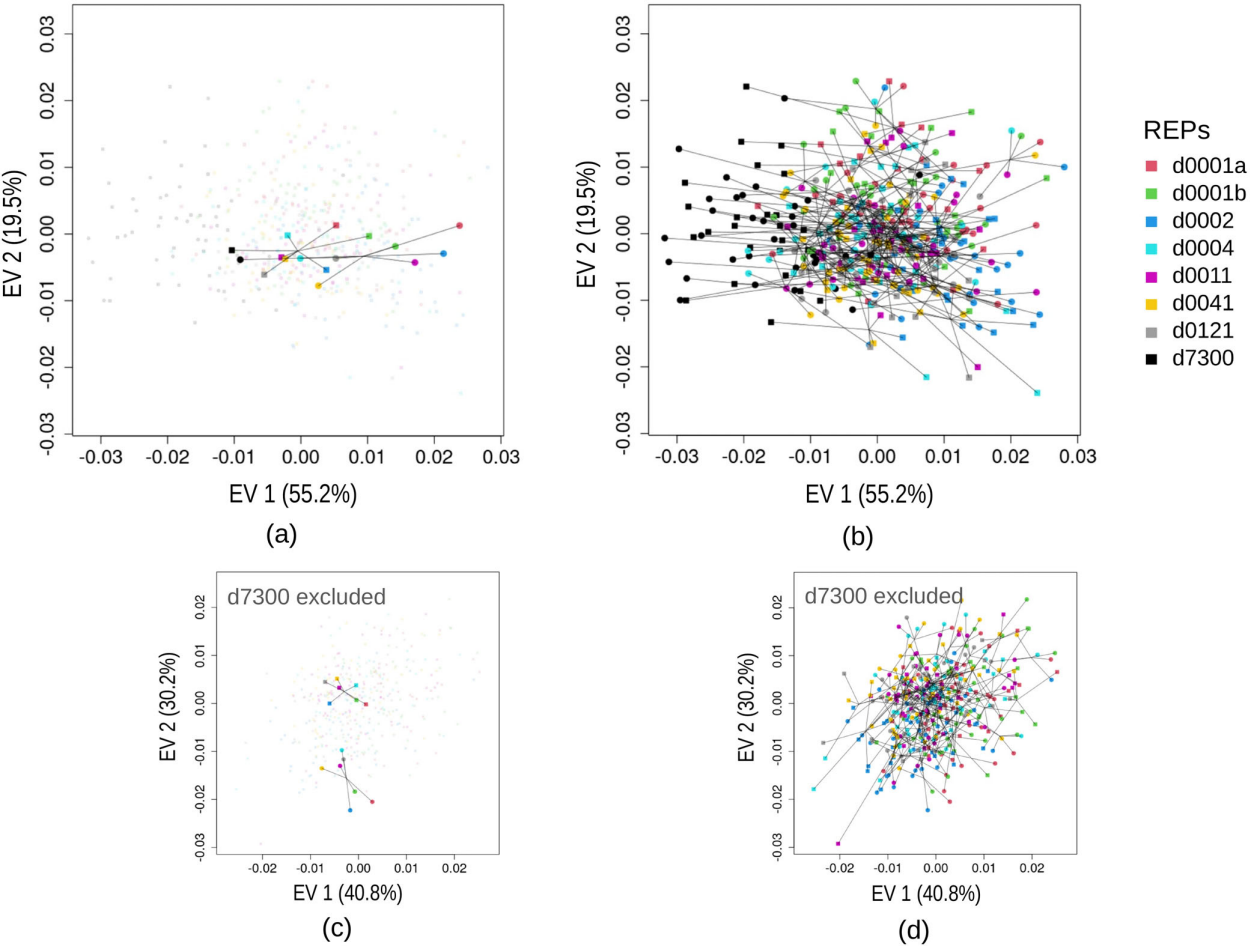
Summary EV plot for the ME ANOVA, using all the landmarks and REPs (filled squares and circles used for males and females, respectively, with different REP colors, as shown in the legend): (a) example showing just two specimens with lines connecting them to their corresponding REP means, (b) same plot with 464 observations, (c and d) same pair of plots as in (a and b) but after removing d7300.

The EV scatterplots (Figure 4), that visualize the main axes of variance due to systematic ME, suggested that d7300 had the largest bias. This was because its sample (black symbols) was separated from those of the other REPs, which overlapped, along EV1 (Figure 4a,b). In this analysis, EV1 dominated the variance pattern in systematic ME, as this axis accounted for almost three times as much variance as EV2. When d7300 was excluded from the ME ANOVA to explore if it was the most impactful REP in terms of bias using all the landmarks, the reduction in Rsq of the systematic ME was modest and the bias remained highly significant (Table 2). However, without d7300, the EV plot became more circular, with EV1 accounting for only 30% more variance than EV2, and no single REP clearly dominated the variation pattern in systematic ME (Figure 4c,d).

**FIGURE 5:**
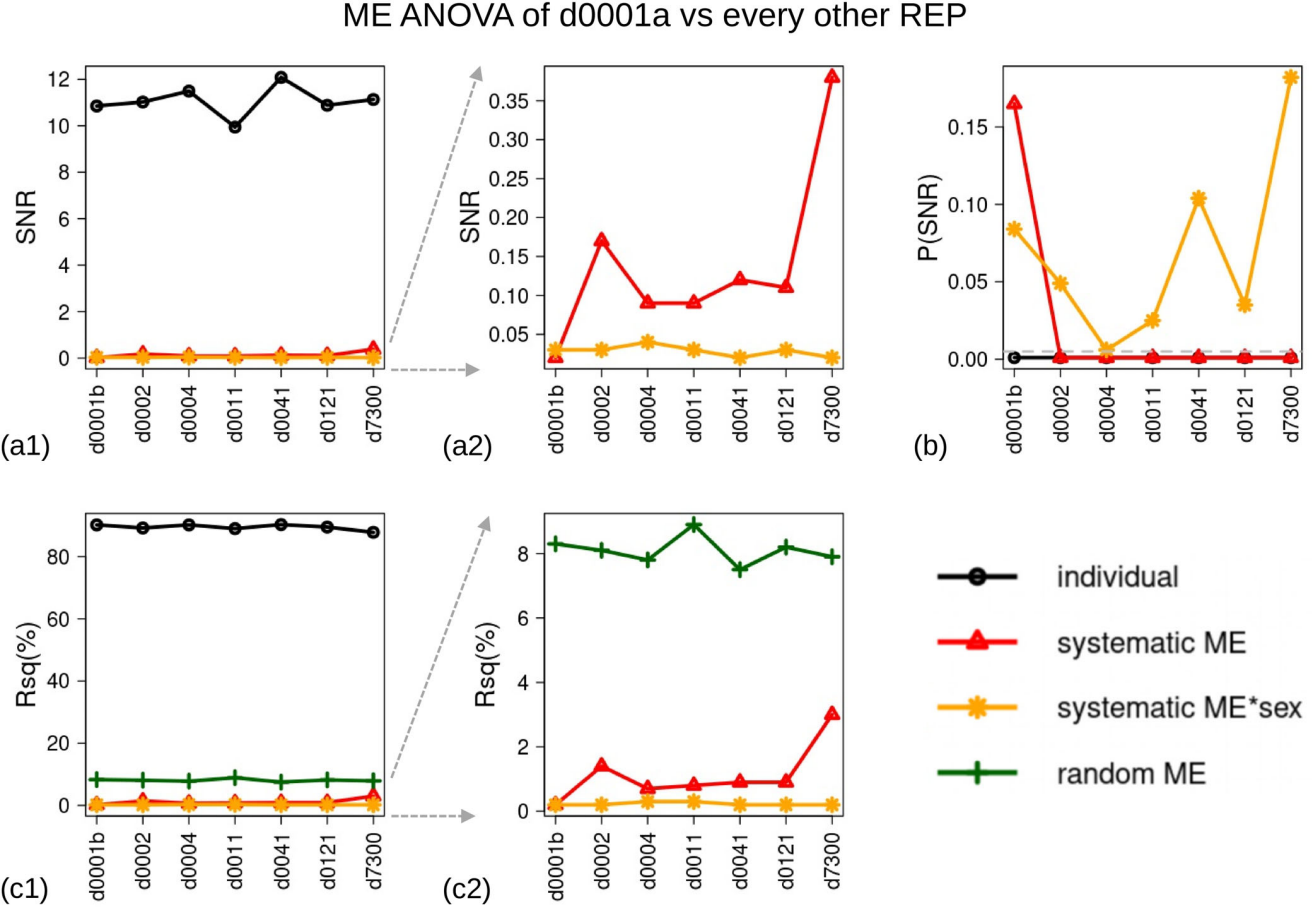
ME ANOVA pairwise between d0001a and every other REP: Profile plots for SNR (a1–a2), their P‐values (b, with broken light gray line marking *p* = 0.005), and the corresponding Rsq (c1–c2). a2 and c2 are the same as a1 and c1, respectively, but they zoom in on factors other than the very large individual factor to appreciate the variability in the different ME components.

Further insight on systematic ME was gained in the pairwise tests (Table 3 and Figure 5) of REP d0001a in relation to each of the other REPs, which are separated from d0001a by progressively longer time lags between digitizations. Including all landmarks, this series of ME ANOVA showed that individual variation (Rsq range = 88%–90%—black line in Figure 5c1,c2) and, therefore, total ME (Rsq range = 10%–12%), which is mostly due to random error (Rsq range = 8%–9%), remained relatively constant despite the increasingly longer time gap between pairs of digitizations. The same happened with the small and negligible interaction between sex and bias (Rsq <0.3%—orange line in Figure 5c2). In contrast, systematic ME (red line in Figure 5) varied in relation to time both in terms of Rsq (Figure 5c2) and SNR (Figure 5a2). It was tiny (Rsq = 0.2%) and statistically negligible when the REPs were done on the same day (d0001a and d0001b). In all other instances (i.e., day 2, day 4, etc.), the bias was significant (Figure 5b). Its magnitude increased sharply in the REP done on day two (d0002 Rsq = 1.4%) but leveled off (Rsq ~1%) in all other REPs between day four and day 141. Finally, in the comparison with the 20 years old REP (d7300), the bias went up to 3% (Rsq), which is approximately three times larger than in the more recent REPs. Thus, for systematic ME, the bias did increase with time as expected. Yet, the increase was not smooth and linear, and the largest effect was the difference between REPs done on the same day, compared to those done between 1 day and 4 months later, and between the “recent” REPs and the oldest one of many years before.

**FIGURE 6:**
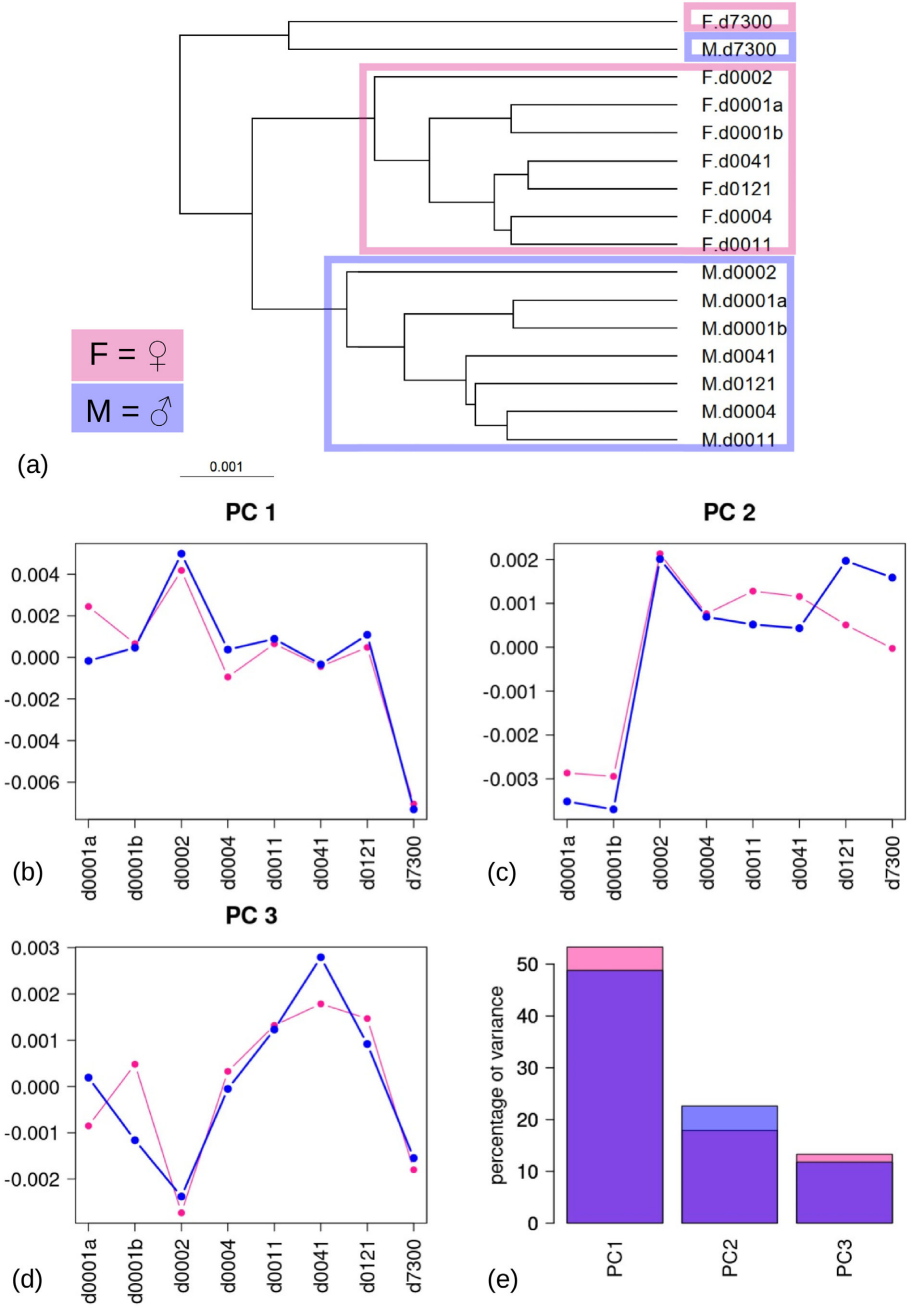
Summary plots for mean shapes of the REPs divided by sex (females = pink, males = blue). (a) UPGMA phenogram, with semi‐transparent pink and blue frames, emphasizing clusters of one or other sex. (b–e) Separate PCAs for females and males, with (b–d) profile plots showing the PC scores of the first three PCs against REPs, and (e) a bar plot showing the variance accounted for by PC1, PC2, and PC3 in females and males.

**FIGURE 7:**
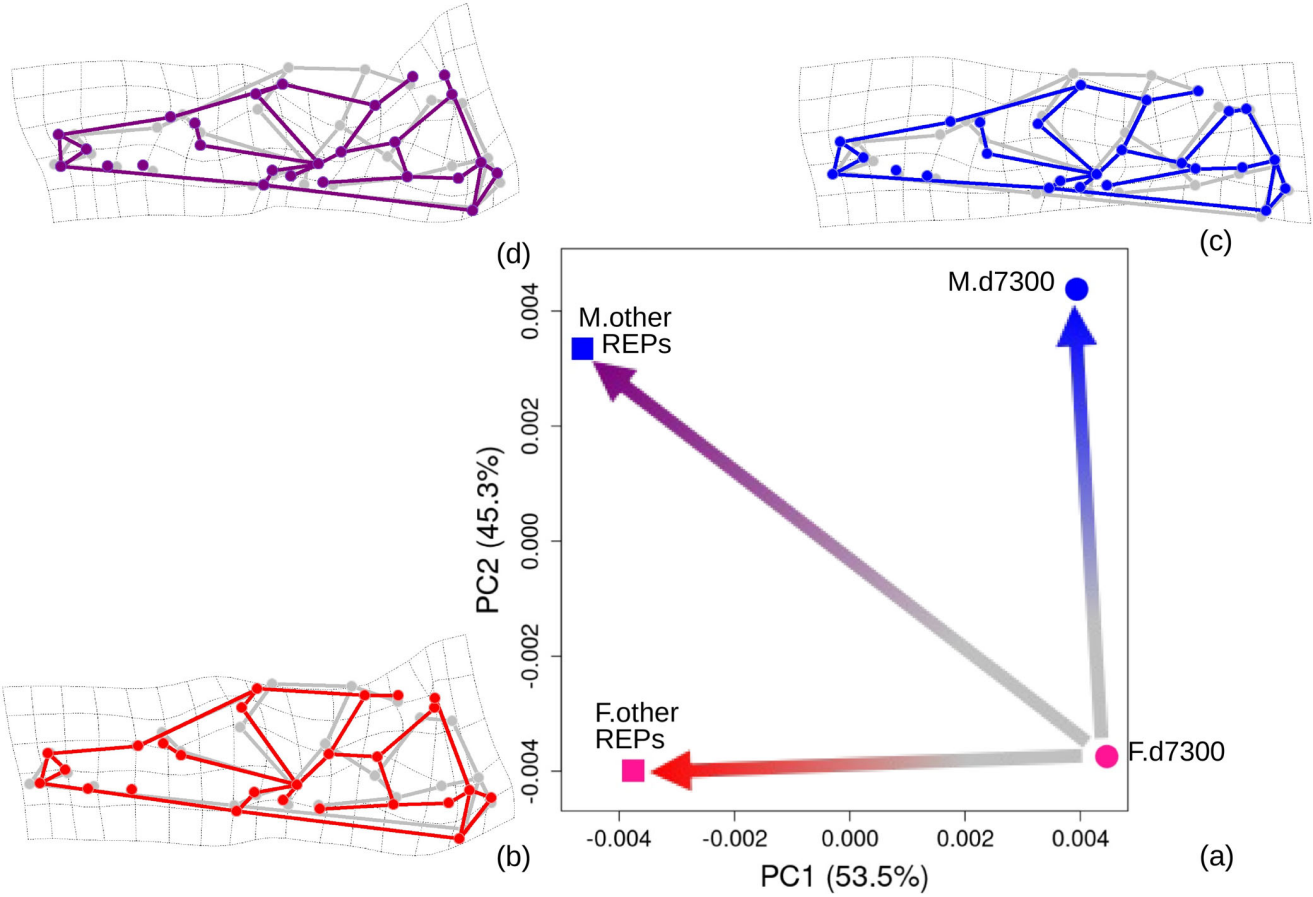
Sexual dimorphism and bias. (a) PC1‐2 (variance in parentheses) scatterplot of average female and male shapes in d7300 versus all the other REPs averaged. PC1 aligns with systematic ME (i.e., mean REP differences) and PC2 with average sex differences. (b–d) Visualization of mean shape differences (magnified 10 times), using, as an example, the d7300 mean female (gray wireframe) as the start shape. The arrows in the PCA scatterplots help stress the direction of the comparisons in the shape diagrams, where the start shape was warped into: (b) the female mean of all other digitizations (red wireframe), (c) the male mean of d7300 (blue wireframe), and (d) the male mean of all other digitizations (violet wireframe). Thus, (b) shows the bias, (c) sexual dimorphism, and (d) the potential bias effect on sexual dimorphism estimate.

The bias was strongest in the REP with a time lag of several years and was confirmed by graphically exploring the mean shape variation including all the landmarks (Figures 6 and 7, with separate mean shapes according to sex and REP). In the phenogram using Procrustes shape distances (Figure 6a), mean shapes clustered according to sex (i.e., all females together with the exclusion of males and vice‐versa), except for d7300. The female and male means of this REP were so isolated from all others that they ended up forming a cluster on their own, although with a branch length similar to those separating females and males in the other REPs. The profile plot of PC1 scores (Figure 6b), which accounted for about half of the total mean shape variance within each sex, showed similar next‐to‐zero scores for all the REPs, except d0002, with a positive deviation from the others, and d7300, with an even sharper but negative deviation. PC2, in contrast, separated d0001a and d0001b from all others and PC3 did not suggest any strongly isolated observation. However, each PC accounted for less than half of the amount of variance summarized by PC1.

Figure 7 visualizes the mean shape differences between sex and REPs. For simplicity, since REPs, other than d7300, clustered together and their systematic ME and the differences were smaller, I averaged them. With the averaged individual data, I computed the female and male mean shapes and compared them with the corresponding mean shapes of d7300. The first two PCs accounted for almost 99% of shape variance, with the four mean shapes approximately forming a square on PC1‐2 (Figure 7). In this scatterplot, PC1 separated the REPs (exemplified in Figure 7b) and PC2, which accounted for only slightly less variance than PC1, separated the females and males (exemplified in Figure 7c). As the distance between the REPs was slightly larger than that between the sexes of the same REP, the plot supported the phenogram's conclusion (Figure 6a) that the bias between d7300 and the others was about the same magnitude as, or even slightly larger than, mean sex differences. The PCA scatterplot also suggested that the differences due to systematic ME were almost orthogonal to those related to sexual dimorphism. In the shape diagrams, males (Figure 7c) had a slightly dolicocephalic ventral cranium compared to females. The bias, exemplified by the REP differences in the two female mean shapes (Figure 7b), strongly affected the zygomatic arch, bulla, and basicranium. Thus, when the female mean of d7300 was compared to the average male shape from all the other REPs (diagonal line in Figure 7d), the mean sex differences were inflated (diagonal of the “square”) and the pattern of sexual dimorphism was slightly distorted (compare Figure 7d with Figure 7c).

### 3.2 R2: Robustness of the findings across REPs including all landmarks

Table 4 reports the results of the permutation tests for the shape variance explained by sex differences, or by static allometry, regardless of sex, and the cross‐validated average HR for sex. In this table, I report for Rsq and HR their 5th and 95th percentiles (highlighted with a dark gray background) to aid the detection of the outliers (emphasized with a light gray background), which, in this case, were REPs whose results strongly deviated from the trimmed 5th‐95th range of all the REPs. The results of Table 5, relevant to this subsection, are those of the total configuration.

**TABLE 4:** Permutation tests of shape variation within each REP and dataset: Sexual dimorphism and cross‐validated classification hit rate (HR) for sex; static allometry with pooled sexes.

				All landmarks			Precise landmarks			Most precise landmarks		
Factor	systME	REP		R2	P	HR	R2	P	HR	R2	P	HR
Sex		d0001a		2.6%	0.079	64%	2.7%	0.074	57%	3.2%	0.067	59%
		d0001b		2.1%	0.238	57%	2.4%	0.116	53%	3.4%	0.034	57%
		d0002		2.1%	0.207	57%	2.4%	0.126	60%	2.3%	0.192	59%
		d0004		2.2%	0.171	53%	2.3%	0.142	60%	2.3%	0.179	57%
		d0011		2.0%	0.255	52%	2.5%	0.093	59%	2.6%	0.148	52%
		d0041		2.3%	0.137	59%	2.5%	0.106	55%	2.6%	0.114	55%
		d0121		2.3%	0.154	57%	2.5%	0.111	59%	2.6%	0.112	52%
		d7300		2.5%	0.106	59%	3.0%	0.035	59%	3.7%	0.039	60%
		5th percentile		2.0%	0.0845	52%	2.3%	0.0465	54%	2.3%	0.075	52%
		95th percentile		2.5%	0.2905	62%	2.9%	0.1364	60%	3.6%	0.745	60%
Simul. example of effect of systME on tests of sex	Small	F_d0001a vs	M_d0001b	3.4%	0.006	72%	2.5%	0.093	57%	3.4%	0.040	57%
		M_d0001a	F_d0001b	2.8%	0.044	66%	2.7%	0.062	57%	3.2%	0.062	52%
	Large	F_d7300 vs	M_other	4.9%	*0.002*	83%	4.7%	*0.001*	74%	4.5%	0.011	66%
		M_d7300 vs	F_other	4.8%	*0.001*	72%	4.4%	*0.003*	62%	3.7%	0.034	59%
Static allometry		d0001a		11.0%	*0.001*		13.3%	*0.001*		17.1%	*0.001*	
		d0001b		11.0%	*0.001*		13.8%	*0.001*		16.7%	*0.001*	
		d0002		10.9%	*0.001*		12.9%	*0.001*		16.2%	*0.001*	
		d0004		10.6%	*0.001*		12.7%	*0.001*		14.5%	*0.001*	
		d0011		9.8%	*0.001*		12.0%	*0.001*		15.4%	*0.001*	
		d0041		10.4%	*0.001*		12.7%	*0.001*		16.2%	*0.001*	
		d0121		10.9%	*0.001*		12.9%	*0.001*		16.2%	*0.001*	
		d7300		12.3%	*0.001*		14.1%	*0.001*		19.5%	*0.001*	
		5th percentile		10.0%			12.2%			14.8%		
		95th percentile		11.8%			14.0%			18.6%		
Simul. example of effect of systME on static allometry	Small	F_d0001a with	M_d0001b	10.3%	*0.001*		12.7%	*0.001*		15.2%	*0.001*	
		M_d0001a with	F_d0001b	11.5%	*0.001*		14.4%	*0.001*		18.7%	*0.001*	
	Large	F_d7300 with	M_other	12.5%	*0.001*		14.6%	*0.001*		19.4%	*0.001*	
		M_d7300 with	F_other	12.0%	*0.001*		14.1%	*0.001*		18.6%	*0.001*	

*Note*: R2 and HR of the ‘within REP’ analyses are summarized using the 5th and 95th percentiles of the observed values (dark gray background). Results outside this 5th‐95th trimmed range are highlighted with a light gray background. The table also exemplifies the potential effect of systME on tests of sex differences and static allometry using two simulated examples, as explained in the Discussion.

**TABLE 5:** Correlations of shape data between pairs of REPs: Summary statistics (# of *r* is the number of pairwise correlations; p10 and p90 are respectively 10th and 90th percentile of *r*, and thus estimate the trimmed range of *r*).

Type of correlation	Dataset	# of *r*	Median	p10	p90
PC1 scores Pearson *r*	All landmarks	28	0.80	0.45	0.90
	d7300 excluded	21	0.76	0.40	0.91
	Precise landmarks	28	0.95	0.92	0.96
	Most precise landmarks	28	0.94	0.93	0.95
Procrustes shape distances Matrix *r*	All landmarks	28	0.79	0.67	0.81
	d7300 excluded	21	0.79	0.66	0.81
	Precise landmarks	28	0.82	0.71	0.84
	Most precise landmarks	28	0.80	0.77	0.82

*Note*: Light gray is used to stress the most important results.

For sexual dimorphism in shape, all the REPs produced congruent results, although d0001a explained slightly more variance and had a slightly larger HR. Nonetheless, none of the tests were significant, Rsq was small (range = 2.0%–2.6%), and HR was only marginally better (57% on average) than a 50% random chance, which was the cross‐validated HR expected for a pair of almost balanced samples with no mean differences.

Also, results of the tests of static allometry using pooled sexes were largely congruent across REPs. Static allometry was always significant with Rsq ranging between 10% and 12%. d0011 and d7300 were, respectively, just below or above the 5th‐95th percentiles for the Rsq of the eight REPs. Yet, these deviations were tiny and made no difference in terms of the main conclusion that there is a moderately large effect of static allometry in adult cranial shape of yellow‐bellied marmots.

Finally, Table 5 shows the results of pairwise correlational analyses between shape data of different REPs. The median correlation of PC1 scores in pairwise comparisons between all REPs was modest, and the trimmed range of correlations was wide (from less than 0.5 to 0.9). Results were similar (in fact, correlations were slightly lower on average, as well as more variable) after excluding the oldest REP (d7300). This indicated that the main pattern of shape variation in PCA scatterplots may vary sharply across REPs. Pairwise matrix correlations between individual Procrustes shape distances of different REPs were also modest (~0.8) but, with a trimmed range of ~0.7–0.8, much less variable than the one found with PC1. Also, the summary statistics of matrix correlations were virtually identical regardless of whether the oldest REP (d7300) was included or not.

### 3.3 R3: Raw coordinates per‐landmark variance and detection of imprecise landmarks

**FIGURE 8:**
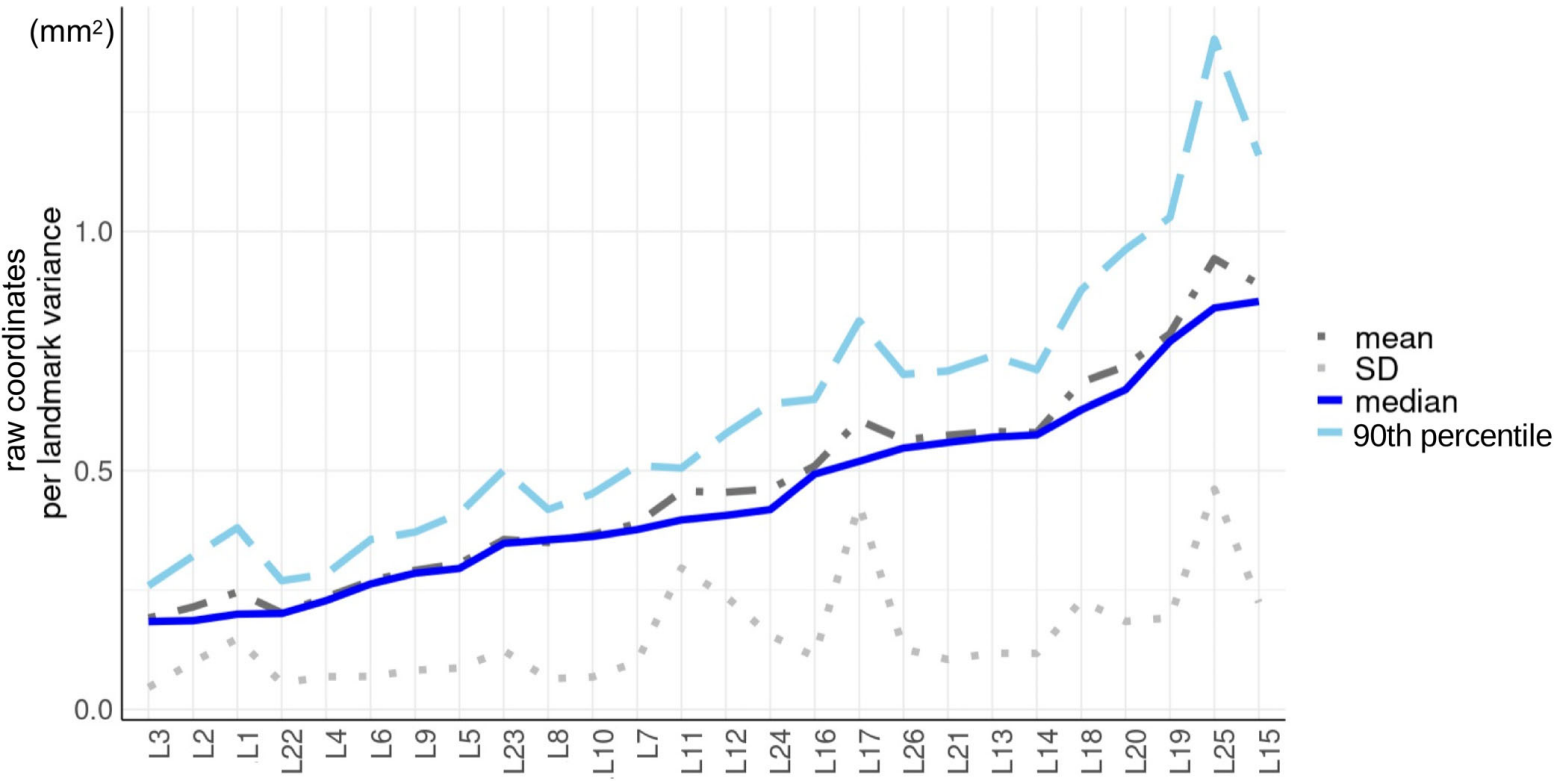
Sample per landmark variances of raw coordinates used to measure absolute imprecision in the digitizations: summary statistics across all eight REPs.

Figure 8 shows the median, 90th percentile, mean, and standard deviation (SD) of raw coordinates per‐landmark variances computed for each individual as a measure of absolute imprecision in the digitization. Means and medians were very similar. I focused on medians in combination with the 90th percentile of individual per‐landmark variances, which summarizes the range of variation after excluding extreme values. Thus, in Figure 8, landmarks were ordered according to increasing median variance. Median variances showed an almost five‐fold range from 0.2 to 0.9 mm^2^. However, the increase in medians from the most (left side of the plot) to the least (right side) precise landmark was gradual. The pattern of variation in 90th percentiles mostly followed that of the medians, thus increasing from left to right in the plot. However, there was at least one sharp deviation: L25 with the second largest median variance, but by far the largest 90th percentile.

**FIGURE 9:**
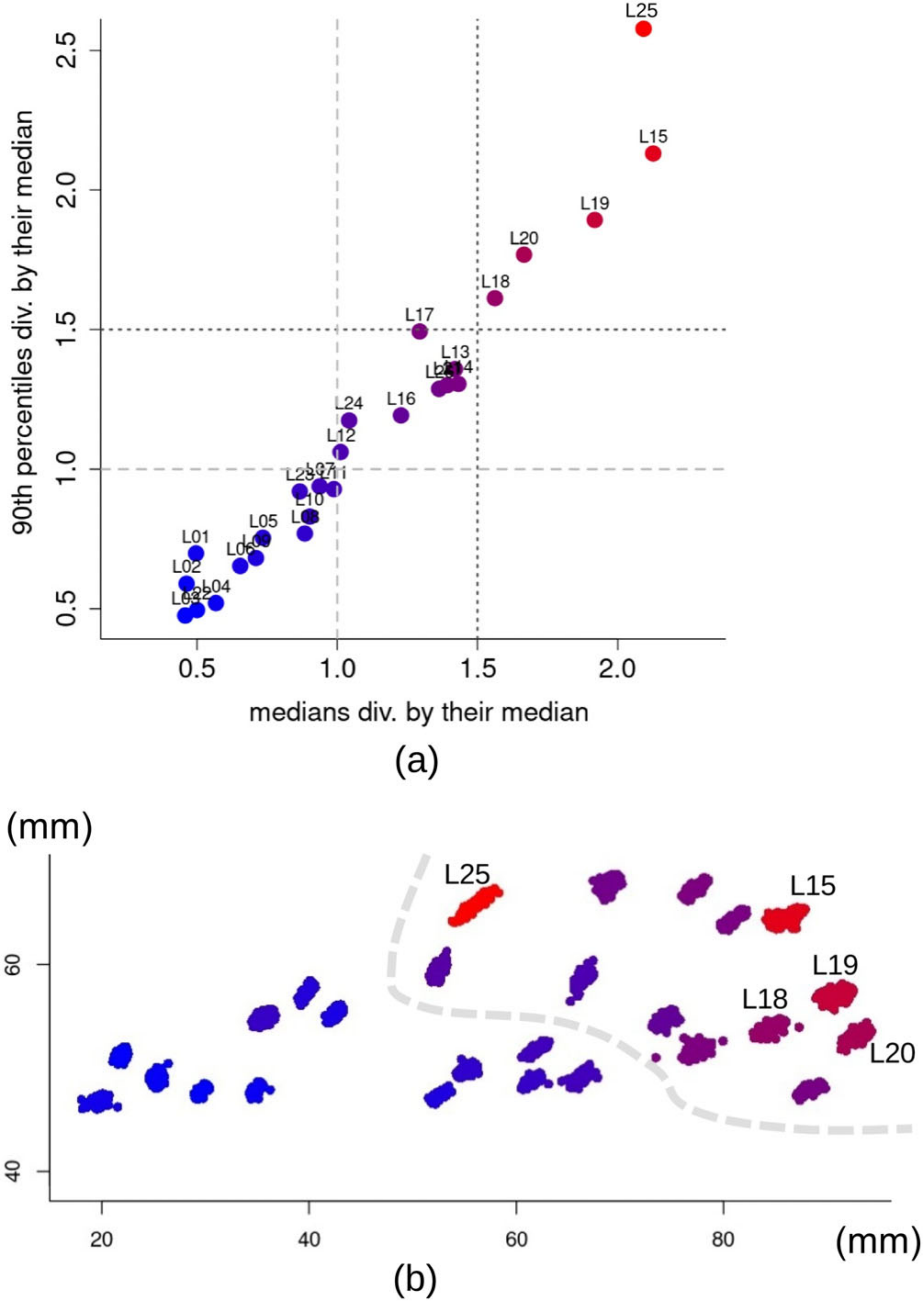
Summary statistics of sample per landmark variances of raw coordinates in the eight REPs. (a) Scatterplot of medians and 90th percentiles of variances (same as shown with blue solid and light blue broken lines in Figure 8), each standardized by their respective median; the light and dark gray broken horizontal and vertical lines at 1.0 and 1.5 detect the least precise landmarks (upper right section of the plot). (b) Absolute per‐landmark imprecision plot showing the deviations of individual raw landmarks from the individual mean location across REPS: The five most imprecise landmarks in (a) are emphasized by their respective numbers, whereas the light gray broken line marks the boundary between precise (below average) and imprecise (above average) landmarks. In both (a) and (b), landmarks are color‐coded, from blue to red by increasing absolute imprecision, with imprecision measured by the sum of the standardized medians and 90th percentiles of raw coordinates per landmark variances (i.e., the values shown respectively on the X and Y axes in a).

In Figure 9a, medians and 90th percentiles of variances are shown after they are “standardized” by their respective median. As explained in the methods, the sum of these values, that is, those on the X and Y axes of the scatterplot, was used as a proxy for the average imprecision of raw landmark coordinates and employed, in both Figure 9a,b, to color code landmarks with a gradient from blue (most precise) to red (most imprecise). Figure 9b is complimentary to Figure 9a and shows the deviation of the raw coordinates of the landmarks of each individual (added to the raw coordinates of an arbitrary individual) from the mean of the eight REPs. Both Figure 9a,b supported the conclusion from Figure 7b that, in relative terms, the least precise landmarks tend to be on the zygomatic arch, bulla, and basicranium (i.e., those to the right of the light gray broken line in Figure 9b). In contrast, the most precise ones were those on the snout.

To obtain a reduced configuration with precise landmarks and one with the most precise landmarks, I, thus, removed respectively the five most imprecise landmarks (L15, L18, L19, L20, and L25, occupying the upper right region of Figure 9a, above 1.5 on both axes) and those five plus another eight with lower than average precision (i.e., all those above 1.0 on the X and Y axes of Figure 9a). For brevity, I will refer to these two configurations of 21 and 13 landmarks as “precise” and “most precise” configurations, respectively. With these, all the main analyses of the previous subsections were repeated (R4) to assess whether removing the landmarks with larger digitization errors helped to reduce total ME and, more importantly, systematic ME.

### 3.4 R4: Assessment of the exclusion of imprecise landmarks on overall ME and bias

In the Procrustes ANOVAs, using the “precise” and “most precise” configurations (Table 2), the results were almost identical to those of the total configuration (same table). However, the magnitude of sexual dimorphism (Rsq) was slightly larger (2.3%–2.5% compared to 1.9% using all the landmarks). Furthermore, in the ME ANOVAs, both the reduced configurations produced results similar to those of the full configuration in terms of statistical significance (significant systematic ME and non‐significant interaction between systematic ME and sex). Therefore, I focused on the ME ANOVAs and specifically on the magnitude of systematic ME, which represented the most interesting component of ME in this analysis. The Rsq of systematic ME using the reduced configurations was slightly smaller (range 1.2%–1.6%) compared to the analysis using all the landmarks (2.2%). The sharpest reduction in systematic ME (Rsq = 1.2%) was, as expected, that of the most precise 13 landmark configuration, although unexpectedly its random ME was slightly larger than that of all other configurations (Rsq ~16% compared ~13%–14%).

**FIGURE 10:**
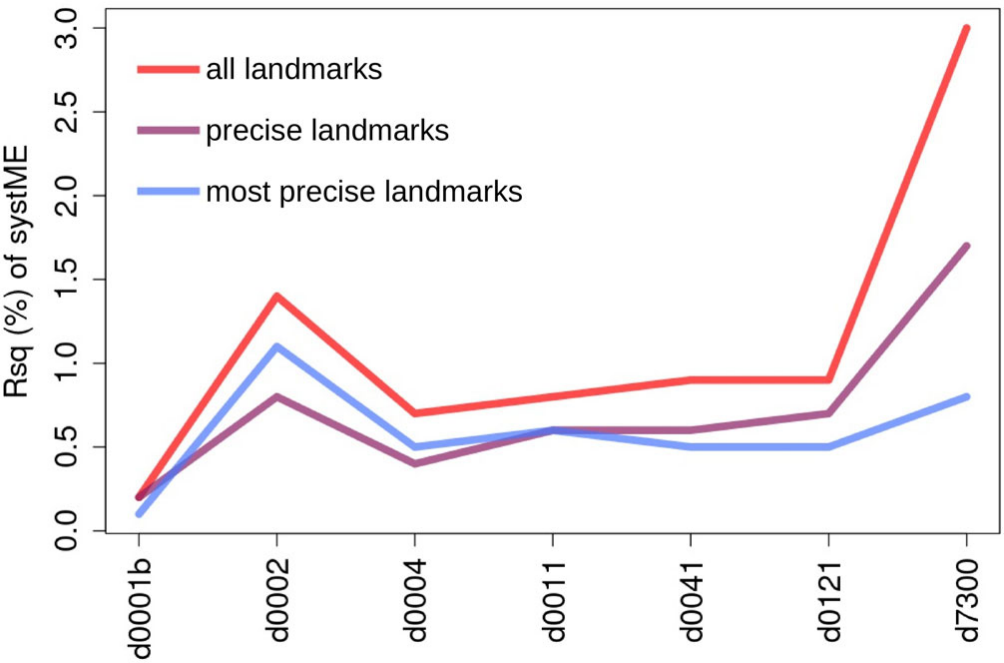
Profile plots of systME Rsq in ME ANOVAs pairwise between d0001a and all the other REPs, using all the landmarks or removing the imprecise ones.

For the reduced configurations, as with the total configuration, pairwise ME ANOVAs comparing d0001a with each of the other REPs are summarized in Table 3. In terms of statistical significance, results were again virtually the same as using all landmarks. Likewise, the most important difference was the magnitude of the systematic ME, which produced a significant SNR always except in the comparison of the two REPs done on the same day (d0001a and d0001b). Rsq for systematic ME in the pairwise comparisons between REPs with progressively larger time lags are shown using profile plots in Figure 10. The trend was very similar in all three configurations except for the oldest REP (d7300): the bias was very small and almost indistinguishable among all three configurations when REPs were done on the same day; however, systematic ME increased sharply on the second day but then slightly decreased and remained approximately the same from day four to day 121. In these five REPs (i.e., from d0002 to d0141), Rsq were usually slightly smaller than using all landmarks (red line) and fairly similar regardless of using precise (violet line) or most precise (blue line) landmarks. The comparison with the oldest REP (d7300), in contrast, showed differences both with the total configuration and between the precise and most precise ones. In REPs of the precise configuration, the bias in the oldest REP was the largest, as in the total configuration, but its magnitude was almost half (Rsq = 1.7% compared to 3.0% using all landmarks). With the most precise configuration, the magnitude of the bias of the oldest REP was halved again (Rsq = 0.8%) and, thus, fell within the range of all other REPs of the most precise set of landmarks (Rsq = 0.1%–1.1%).

The tests of mean sex differences within each REP, using the reduced configurations, are shown in Table 4. The results were similar to those of the total configuration, with REPs being generally highly congruent in terms of the small Rsq (2%–3% with “precise” landmarks and 2%–4% with “most precise” ones) and the non‐significance of this effect. However, in both datasets, d7300 had an Rsq slightly above the 95th percentile. Accordingly, cross‐validated average HR were barely above random chance (on average slightly below 60%). Congruence was high also in the tests of pooled‐sex adult static allometry (Table 4). As in the total configuration, they were always highly significant. Rsq were slightly larger (on average, 13%–16% compared to 11% in the total configuration). The deviations from the main range (5th–95th percentile) of Rsq were minor, except d7300 in the “most precise configuration” whose Rsq = 19.4% was fairly above the 18.6% 95th percentile for this configuration.

Removing imprecise landmarks had a more evident impact on correlational analyses, with very similar results for both the “precise” and “most precise” configurations (Table 5). For PC1 scores, the median correlation was ~0.95; thus, it was larger than the 0.8 found using all the landmarks. Moreover, the range of variation was narrower, with all pairwise correlations consistently >0.9. Thus, removing imprecise landmarks increased the repeatability of the PC1 scores. In contrast, with matrix correlations using Procrustes shape distances, r was about the same as in the full configuration (~0.8). However, the range of variation in matrix correlations was smaller, and this reduction was slightly more pronounced using the “most precise” landmarks (range 0.77–0.82).

## 4 DISCUSSION

### 4.1 Systematic ME and time lags in data collection: The “visiting scientist effect” is real but may have little impact

The overall ME pattern in the repeated digitization of yellow‐bellied marmot ventral cranial landmarks proved that time lags in landmark digitization can introduce a small (Rsq ~1%–3%) but non‐negligible bias in Procrustes shape data (Tables 2 and 3). The other important questions in the study were whether the bias increased with the duration of time lags and what its impact was on the results of group comparisons (sex differences, as a within species example) and the analysis of shape in relation to a covariate (within species static allometry in the current study), as well as in terms of congruence between shape datasets (correlational analyses). In the next paragraphs, I summarize and discuss the main results of the extensive series of analyses I did, before drawing the main conclusions and suggesting some potential implications and future directions in the broader context of the impact of ME in GMM research.

When the first REP, done in day one, was compared pairwise with each of the other REPs, individual variation was rather large, random ME was of moderate magnitude, and both remained relatively constant in all comparisons. In contrast, in the same pairwise tests, systematic ME varied and was always significant, except when REPs were done in the same day, although it was much smaller than random ME. Also, systematic ME was similar (non‐significant interaction and tiny Rsq) in females and males. Using all landmarks, when time lags between REPs were relatively short (from 1 day to a few months—Table 3), the bias was modest (Rsq ≈1%). With a much longer time lag of many years, however, there was a three‐fold increase in magnitude for the systematic ME (Rsq = 3%). A closer inspection of the Rsq values for the total configuration in Table 3 reveals that this sharp increase from 1% to 3% made the total ME slightly larger (12% in the comparison with d7300 instead of the average 10% found with other REPs). This increase in ME using the oldest REP caused a concomitant albeit, in relative terms, tiny reduction in individual variation (down to 88% from an average of 90% in other pairwise comparisons). Because the change in magnitude of individual variation was very small, however, individual differences always remained significantly larger than ME. Yet, the time‐related bias in the oldest d7300 REP was strong enough for this REP to dominate the pattern of variation in systematic ME (Figure 4a,b).

Finding a stronger bias, using d7300, does not imply that this is the only cause of non‐negligible systematic ME in the dataset. The two REPs done on the same day were virtually unbiased, but all the other REPs, despite lacking time‐related increase in systematic ME, showed significant biases. This was demonstrated by the pairwise comparisons with the first REP of day one and the observation of a significant bias, although smaller (Rsq = 1.4% compared to 2.2% including all the REPs), even when the oldest REP was excluded from the ME ANOVA with all the landmarks and all the other REPs (Table 2). Overall, these findings indicate that there is a “visiting scientist effect” on ME in the study dataset. The effect operates mainly on systematic ME, which increases even after a very short time lag (1 day) and is consistently present. With much longer time lags, the bias becomes significant and slightly larger than the magnitude of sexual dimorphism, a small but biologically interesting component of shape variation in yellow‐bellied marmots (Cardini, 2024b; Cardini & O'Higgins, 2004).

Although I only explored landmark digitization error, time‐related biases may be aggravated by other sources of ME, such as, for example, the orientation of a specimen when positioned for a photograph, the use of a different camera, and the possibility that data are collected by multiple operators over a long time span (Arnqvist & Martensson, 1998; Fruciano, 2016). As I discussed before (Cardini, 2024a), in my experience on mammals, the simple repositioning of a structure, such as a mandible or cranium, can introduce an additional amount of ME of comparable magnitude to the total ME of landmark digitization. If that happened in a systematic fashion, such that, after a relatively long interruption in data collection, a subtle but consistent difference in orientation biases the photographs (or, for 3D data, the view under which 3D landmarks are placed), there is little doubt that systematic ME would be larger. Likewise, there is evidence that multiple operators, because of small directional variation in how they digitize landmarks, can bias Procrustes shape variables so strongly that the data become unusable (e.g., Daboul et al., 2018).

Interestingly, even when a single source of error is considered, as in my study, the relationship between time lags and the magnitude of the bias in the digitization is not simple and linear, but complex. However, because REPs only concerned digitization error, it was possible to assess what landmarks had the lowest precision by computing per‐landmark variances of raw coordinates in the REPs of each individual.^3^ Using precise and most precise reduced configurations, the impact of removing imprecise landmarks was small in terms of total ME. For instance, in the Procrustes ANOVA, total ME accounted for 17% of shape variance using the most precise landmarks versus 15%–17% using the precise and total configurations. However, the ME ANOVAs of the precise and most precise subsets of landmarks showed an impact on systematic ME. The bias was still present, but smaller. This reduction was enough to make higher and less variable the correlations of shape data between REPs. The effect was particularly evident on PC1. Because random ME was almost unchanged in the reduced and more precise configurations, this observation suggests that the bias specific to highly imprecise landmarks may be picked up by the main PCs. This observation, if confirmed in future studies, is worrying, as the first PCs are typically used in scatterplots for interpreting results and, sometimes, for dimensionality reduction in statistical tests.

In pairwise comparisons (Table 3), using the most precise landmarks in terms of absolute digitization error largely removed the effect of the longest time lag and made its systematic ME about ¼ of that found in the total configuration. That a careful selection of high precision landmarks reduces ME has already been observed (e.g., Fruciano et al., 2017). This is good news when a researcher is interested in very small differences in shape that are more strongly impacted by biases. However, higher precision came at the cost of halving the number of landmarks on yellow‐bellied marmot crania and completely missing the region between the zygomatic arch and the cranial base, where the most imprecise landmarks tended to be.

That all highly imprecise landmarks occurred in the posterior region of the cranium raises the question about why they specifically tended to be there. One can only guess, and reasons can be multiple and complementary. The posterior region of the ventral cranium is complex and has several landmarks on curves (e.g., L24‐25), which are almost by definition difficult to precisely locate and, thus, might add some random noise. That complex anatomical structures can be more prone to digitization error, especially in photographs, has already been suggested (Vrdoljak et al., 2020). It is also not unlikely that an operator digitizing less well‐defined landmarks, such as those on curves, after a time lag, tends to see them consistently in a slightly different place (e.g., slightly lower than before), thus introducing a bias. Other imprecise landmarks occurred on large foramina, where it is easy to digitize a landmark slightly off its previous position in a different digitization session. Besides, foramina, and sometimes sutures (such as the one between the basisphenoid and basioccipital), in the caudal region of marmot ventral crania were not always easy to see, either because of bone fusions or because of the lower quality of the photograph in this complex part of the cranium. That was also the case, likely, with the tips of the external auditory meatus, which are ill‐defined, sometimes broken, and whose boundaries might be fuzzy in the images. Some of these issues (e.g., when sutures are fused) have no simple solution. Others, such as the quality of the photograph, can be improved using higher resolution cameras and a better illumination.

### 4.2 Is the “visiting scientist effect” truly negligible? Exploring the issue by simulating an extreme but plausible scenario in studies of population differences

Statistical significance with a small effect size, as it happens with systematic ME in this study, can be a useful warning especially when the effect being tested is small, as it happens for sex differences in the shape of yellow‐bellied marmots. Yet, although there was also an effect of ME on PC1 (and likely on most of the other PCs), one might argue that the bias did not alter the main conclusions of analyses of the “biological” effects (sex but also allometry) when these were tested in the full shape space. Although it cannot be excluded that random ME, which was similarly large in all REPs, reduced statistical power, it is good news that the impact of systematic ME was overall negligible not only using precise configurations but also with the total configuration. Thus, in all REPs sexual dimorphism was small and did not reach statistical significance. In contrast, static allometry was larger after excluding more imprecise landmarks but similar in magnitude across REPs and significant all the time, although possibly with an inflated effect size in the oldest REP, which was consistently above the 95th percentile of the eight REPs.

Can we then conclude that, in this specific dataset, when the researcher is interested in sex differences and static allometric variation, a small but significant systematic ME should not be a cause for concern? If that was the case, the “visiting scientist effect” in my dataset would be real but its impact negligible even for a tiny effect such as sexual dimorphism in the cranial shape of yellow‐bellied marmots. But is that true? Some interesting insight comes from the results of a small set of exploratory simulations of a fairly extreme scenario (but, as I argue below, not unlikely in taxonomic studies). The scenario is one in which females were digitized first and males only some time later. This would clearly be a flaw in the design of data collection, where randomization of the specimens in a sample is highly desirable. It is also a potential flaw, which is easy to avoid most of the time using landmarks digitized on photographs. With photographs (2D images or 3D scans), assuming the photographic or scanning settings are well standardized, a researcher can first acquire all the images and only later, within the shortest possible time span, digitize the landmarks in a single session on all individuals. This would minimize any potential bias related to time lags. However, if the researcher is collecting data with a 3D digitizer in different species by visiting several museums, as I described in the Introduction, the digitization will likely have inevitable time lags, as one may be forced to measure first one taxon, which was well represented in a museum, and only later a different one, unavailable in the first museum but present in a different one.^4^ Indeed, it is not uncommon that museum collections are biased in taxonomic composition. In Europe, many mammal collections in the largest natural history museums tend to have a much better representation of species and specimens from former colonies. Other species, or conspecific individuals, whose range was outside the colonies, may be poorly represented or completely absent. Thus, as said, it may be inevitable that most or all individuals of species A are collected first, and those of species B only later. A lack of funds (also not uncommon in basic research in museums) may even extend over several years the span of the data collection done by a visiting scientist for a specific study. Thus, time lags in data collection are often a reality for visiting scientists working in museums.

Now that this potential issue has been better explained, I go back to the small simulated examples, where I tested again sex and allometry, but I simulated the problem of biased data by sampling females of one REP together with males of a different REP. Specifically, I chose for the first example the two REPs done in the same day (d0001a and d0001b), as they have the smallest bias and any potential problem should be small or totally negligible. For the second example, I averaged individuals of all REPs other than the oldest (d7300) and compared them with the oldest REP (with the same justification as for the visualization of shape mean differences in Figure 7). Since the oldest REP had a much larger bias compared to the others, I predicted a stronger effect on tests of sex differences and static allometry when females of d7300 were analyzed together with males (averaged) of other REPs, and vice versa. As in the main analyses, both simulated examples were done using all landmarks, as well as the precise and most precise configurations.

Results are reported in Table 4, so that they can be directly compared with those of within REP analyses (described in 3.2–3.4). When the bias was small (REPs of the same day), the magnitude of sexual dimorphism was appreciably overestimated (above the 95th percentiles of the results of within REP analyses reported in the same table and highlighted with a dark gray background), and in one case using the total configuration, very close to significance. Using precise landmarks, however, the effect of the bias almost disappeared, and results were again congruent with those found within each REP. When the bias was large, however, which is when females of the oldest REP were analyzed with males (averaged) of other REPs, and vice versa, the consequences of the systematic error were much more pronounced. Rsq for sexual dimorphism was about twice the average Rsq of analyses done with females and males of the same REP, and both the total and precise configurations (but not in the highly precise) reached significance, with cross‐validated HR above the 95th percentiles of the within REP results.

In the analyses of static allometries, the main difference in the simulated examples, compared to the analyses done with females and males belonging to the same REP, was a general trend toward overestimating effect size (Rsq), which was more pronounced in the total and precise configuration when the simulated bias was large (oldest REP versus the other REPs averaged). That the impact of the simulated bias was weaker in the analyses of static allometry is unsurprising. Static allometry was significant in all analyses and had a much larger effect size (Rsq = 11%–16% on average, which is approximately four times larger than sex) and, therefore, even a bias accounting for 1%–3% of variance could not substantially change the outcome of the regressions. With sex, in contrast, the magnitude of the bias was close to the magnitude of the effect being tested (on average ~2%–3%), and thus had an impact strong enough to change the conclusion of the analysis from that of a small and generally negligible sexual dimorphism (as it is usually the case with marmot morphology—Cardini, 2024b) to one of apparently larger and highly significant sex differences in cranial shape.

It is also interesting to observe that the overestimation of sex differences, especially when the bias was larger in the simulation, happened despite the differences due to the systematic ME being almost orthogonal to mean sex differences. Orthogonality is suggested by Figure 7, with the bias picked up by PC1 and mean sex differences by PC2, which are statistically uncorrelated to PC1. This plot also indicates the reason for the overestimate. Since the four mean shapes (oldest REP and average of the other REPs, split by sex) are approximately in the vertices of a square in the space of PC1‐PC2 (99% of shape variance), the mean difference between females of one REP and males of the others (and vice versa) corresponds to the distance along the diagonal of the square. Mean sex differences, when females and males belong to the same REP, are, in contrast, measured by the (approximately) vertical side of the square. Since the diagonal of a square is always longer than the side, it is, thus, inevitable that the bias using females and males of different REPs causes sexual dimorphism to appear bigger. Thus, contrary to an earlier suggestion I made (Cardini, 2024a), that a systematic ME is really concerning if it happens in the same approximate direction of the shape differences a researcher is interested in, because it may inflate or deflate the size of the effect, a bias may lead to inaccurate conclusions even when it is orthogonal to the biological aspect of shape variation being investigated.

If even a very small bias can impact the outcome of tests of small group differences, such as those of sexual dimorphism in adult marmot cranial shape, one might wonder whether, contrary to my statement in the Introduction, we can really be confident that the same cannot happen with CS. The ME ANOVA, as currently implemented in geomorph‐RRPP and described in Collyer and Adams (2024a, 2024b), is specific to landmark coordinates and, thus, cannot be used for testing univariate size. However, one can have clues about the impact of the small differences between REPs by comparing the average REP differences in females and males with the between‐sex median differences. In females, depending on the REP, the median ranges from 132 to 135 mm, and in males, it ranges from 135 to 138 mm. Median sex differences within REP vary between 3 and 4 mm. Thus, as with shape, the magnitude of the bias in cranial size can be similar to the magnitude of the biological effect being tested (sex differences, in this example). A repeated measure ANOVA using all eight REPs is highly significant in both females and males (*p* < 0.005). This type of ANOVA differs from the ME ANOVA (see Collyer & Adams, 2024a, 2024b), but it may offer an alternative to explore biases if the ME ANOVA is not extended to univariate data. As with the shape data, significance may be an important warning, but one should also compare the effect size of the bias with that of the biological factor being tested for clues about possible impacts. A simulation similar to the one I used for shape can also offer clues to better appreciate the consequences of a bias on comparisons of CS among groups. For instance, if the REP with the highest average cranial size for females and the lowest for males had been compared, the test for sexual dimorphism in cranial size among adult yellow‐bellied marmots would have shown no significant difference (*p* = 0.174). If the same test was done within REP, however, it would have been marginally significant (*p* = 0.02, i.e., below 0.05, although above the 0.005 used in this study).

I have already stressed that the scenario I simulated, where groups are compared, which were each digitized at different times, is extreme. Yet, it is not unrealistic in extensive data collections of many populations and species across several museums, which is why I nicknamed it the “visiting scientist effect.” Most of the time, one could avoid the issue with a careful design of the data collection, but sometimes this might be impossible. Time lags happens when data are collected across multiple museums and institutions, but they might occur in a variety of other cases. An interesting example is the acquisition of morphometric data on live animals in the field. For instance, Djurakic and Milankov (Djurakic & Milankov, 2019, 2020) collected genetic and morphometric data on *Testudo hermanni* across dozens of localities in the Balkans. Animals were captured in the field, blood samples were obtained and individuals were photographed to quantify carapace and plastron geographical shape variation. It is inevitable that such an extensive data collection requires months or even several seasons of field work. Even if landmarking is done after randomizing specimens, when all populations have been sampled, there might be small unintentional differences in how the photographs were taken in one or the other localities. Light conditions may vary, a camera might get damaged and need replacement, and standardized settings may be difficult to maintain with live animals that must be quickly released to minimize stress from capture and handling. Besides, unlike in studies of museum specimens, the variable conditions of data collection in the field are difficult to simulate to assess systematic ME. Re‐training carefully and standardizing the protocol for data collection as much as possible may, therefore, be the best a researcher can do in these cases to avoid or minimize potential biases.

There is another couple of points that are important to emphasize in relation to the effect of time lags. The first is that systematic ME showed a stepwise trend. It was almost imperceptible in the second REP, done in the afternoon of day one, compared to the first REP, done in the morning. Then, the bias increased sharply on day two and remained approximately the same up to 4 months later. Finally, it raised sharply again in the REP of many years ago. As with other findings, whether this is generalizable beyond the current study will have to be assessed by future research. However, one might wonder what happens when time lags are intermediate between several months and many years. The lack of REPs with a time lag of, say, 1 or 2 years is a limitation of my analysis. However, I hope future, longer‐term studies with repeated digitizations over several years will address this.

The second point I want to emphasize is the sharp increase in systematic ME in the digitizations performed on the second day compared to those done on the first day. The bias on the second day is similar to, but actually slightly larger than, that in REPs with longer time lags (with the exception, unsurprisingly, of the 20‐year‐old REP). That after 1 day the bias is larger than after weeks or months is counter‐intuitive. The reason could simply be chance, but it is also possible that part of the imprecision was due to the operator's fatigue. In this article, I am focusing on digitization bias and the “visiting scientist effect” using a within species study case. In a follow‐up paper, I examine this issue at the between‐species level. This next study includes the yellow‐bellied marmot dataset, along with REPs conducted with the same design on four additional marmot species. Since data for all species were collected in the same rounds of repeated digitizations, by the end of day one, I had digitized over 11,000 landmarks in total. It is quite possible that, after this considerable effort, I was tired on day two, leading to reduced precision. If this guess is correct, there is another important caveat, that morphometricians should bear in mind: it is probably unwise to digitize too many specimens per day, as precision might be affected both within that day (e.g., in specimens digitized toward the end of the working day) or in the next days. Practicing before the main data collection begins may help to assess how many specimens an operator can digitize in a day before one starts losing concentration, is less precise and fatigue increases so much that even the day after he/she feels tired of landmarking.

### 4.3 Conclusions

The traditional framework that largely relied on the Procrustes ANOVA, comparing individual variation to total ME, might lead to overoptimistic conclusions, since individual variation generally tends to be much larger than ME and, thus, it can mask a subtle effect of systematic ME (Collyer & Adams, 2024a). On the other hand, however, the very powerful ME ANOVA might be over‐sensitive to a small systematic ME, since the bias is related to random noise. Indeed, the approach could be probably modified to compare systematic ME directly to the effects being tested, such as sex differences or static allometry. Nonetheless, even in its current form, the ME ANOVA provides a useful warning that there might be problems. Thus, in the context of a possible “visiting scientist effect,” a researcher might carefully include potentially inevitable time lags in the design of the repetitions used to estimate ME and fruitfully employ the ME ANOVA to test systematic ME. For the assessment to be accurate, one will have to simply remeasure a representative subsample before any new round of data collection in order to compare it with previous, older, REPs (Evin et al., 2020).

If the ME ANOVA suggests a non‐negligible bias, a morphometrician should be cautious and carefully explore its potential impact. More research is clearly needed. In my dataset, for instance, I showed that systematic ME had a largely negligible impact on estimates of static allometry. However, that same bias could appreciably alter the conclusions of the test of sexual dimorphism in shape, if a time lag in the data collection had biased the sample composition, so that most females were measured first and most males only later (or vice versa). To minimize the risks of impacts, as a consequence of the “visiting scientist effect,” the operator could re‐train her/his landmarking skills before doing more data collection. The researcher could also explore whether a subset of more precise landmarks can be designed, which adequately measures the morphological aspects one is interested in but also minimizes biases. If nothing really works, as neither re‐training or using a reduced configuration allows to control for the bias, it is clearly bad news. One might have to discard some of the data, abandon the study or, if feasible, change the aims. Yet, the researcher has gained crucial awareness of the problem and may, thus, avoid expensive further rounds of data collection, that would lead to flawed analyses and the potential publication of inaccurate findings.

## AUTHOR CONTRIBUTIONS

**Andrea Cardini:** Conceptualization; investigation; funding acquisition; writing – original draft; methodology; validation; visualization; formal analysis; data curation.

## Endnotes

^1^
Throughout the paper, I will use the terms bias, directional ME, and systematic ME interchangeably to refer to consistent inaccuracies in measurements, specifically the Cartesian coordinates of a landmark in this study.

^2^
The plot of raw per‐landmark deviations suggests an example to make less abstract the definition of random and systematic ME. If, say, in the oldest REP (d7300) I systematically tended to digitize L15 to the right and colored L15 in this REP in black, using orange for this landmark in all other REPs, I should notice an elongated scatter where the black landmarks are slightly separated from the orange ones to their left. This would suggest a bias in L15. I did not use this type of plot simply because there tends to be so many overlapping points that it is hard to discern the colors (unless one zooms in each specific landmark). It is also useful to bear in mind that random ME in the plot of REP deviations in raw coordinates does not have to be circular (isotropic). It could be random (with no REP having landmarks consistently to the left or right etc. of those of other REPs), but, for instance, elliptical.

^3^
I stress again that this would not be possible if other sources of error were included (which is desirable for an accurate assessment of ME—Arnqvist & Martensson, 1998; Fruciano, 2016). For instance, if photographic errors, when a specimen is repositioned and a second photograph is taken, had been included, per‐landmark variances including both photographs of each specimen would be meaningless, because the position of the cranium in the photographs is no longer constant. One could still try to have clues on landmark precision after Procrustes superimposing the specimens. However, that would be inaccurate and potentially misleading (Cardini & Verderame, 2022). As an extreme and unrealistic example, that nonetheless makes the issue more intuitive, even if all the ME was in a specific single landmark, after the superimposition this ME would be spread (probably dishomogeneously) across all the landmarks in the configuration. Likewise, the opposite can occur when ME in the raw coordinates is similar across all landmarks but appears to vary in the shape coordinates after performing the Procrustes superimposition. Thus, one may still have some hints on per‐landmark variation in REPs, and explore if removing the apparently most variable landmarks in shape have an impact on ME, but he/she must be aware of the inaccuracy of the approach.

^4^
Even with photographs, in fact, one may lately discover a rare sample of interest (or have the funds for another expensive visit) only after having already digitized the landmarks in its main study sample.
